# Continuous Insulin Infusion System and self-care in Type 1 Diabetes Mellitus: scope review

**DOI:** 10.1590/1518-8345.8001.4852

**Published:** 2026-06-15

**Authors:** Raquel Rodrigues da Costa Brilhante, Carla Cristina de Sordi, Paulo Weslen Carneiro Gonçalves, Vanessa de Araujo Lima Freire, Amelina de Brito Belchior, Maria Luiza Pereira Costa, Raquel Sampaio Florêncio, Sherida Karanini Paz de Oliveira

**Affiliations:** 1 Universidade Estadual do Ceará, Fortaleza, CE, Brazil.

**Keywords:** Insulin, Insulin Infusion Systems, Self Care, Self-Management, Knowledge, Diabetes Mellitus

## Abstract

**Objective:**

to map the evidence available in the literature on self-care among people with type 1 diabetes mellitus using continuous insulin infusion systems.

**Method:**

this is a scoping review based on the principles recommended by the JBI and the PRISMA guidelines for data extraction, conducted using ten databases and gray literature. The PCC strategy was used for data collection. Studies were selected after duplicates were removed and individual and paired assessments were conducted. Descriptive analysis and diagrams were used to present the results.

**Results:**

an analysis of the 18 selected articles showed three self-care behaviors: glycemic management, insulin bolus, and carbohydrate counting. The factors influencing these behaviors were Knowledge, Freedom/flexibility, Sensor use, Context, and Responsibility.

**Conclusion:**

the studies indicate that in-depth knowledge of self-care enables professionals to make better decisions about individualized treatment plans and enables people using the Continuous Insulin Infusion System to seek actions and information related to daily care for proper diabetes management. There is a need for future intervention studies to optimize self-care in this population.

## Highlights

**(1)** Identifies the self-care behaviors of people with DM1. **(2)** Analyzes the factors that influence self-care behaviors. **(3)** Helps professionals make decisions regarding individualized treatment plans. **(4)** Assists professionals in seeking actions and information related to daily care.

## Introduction

Type 1 Diabetes Mellitus (DM1) is an autoimmune condition characterized by the destruction of beta cells in the pancreas, usually leading to absolute insulin deficiency^1^. Although it is the most common type of diabetes mellitus in children and adolescents, studies indicate that there are more new cases of DM1 diagnosed in adulthood than in childhood and adolescence^2^.

In 2022, it was estimated that 8.75 million people worldwide had DM1. Brazil was among the 10 countries with the highest prevalence of DM1 across all age groups^3^. This epidemiological scenario requires strategies that encourage self-care practices to achieve satisfactory therapeutic results and prevent complications. Self-care practices include meal planning, physical activity, regular use of medications, especially insulin, and routine capillary blood glucose monitoring^4^.

For satisfactory glycemic management of DM1, intensive insulin therapy that mimics physiological insulin secretion is emphasized^5^. Among these treatments, the Continuous Subcutaneous Insulin Infusion (CSII) is an electronic device that continuously infuses insulin into the subcutaneous tissue, mimicking what occurs in the body of a person without diabetes to achieve euglycemia between meals and release insulin at mealtimes. The use of CSII can provide greater safety and autonomy in the treatment of DM1^6^.

To use CSII, people with diabetes need to acquire and develop knowledge and skills for appropriate treatment care. This requires an interprofessional approach focused on diabetes education for patient care. Diabetes devices, such as CSII, are complex machines that rely heavily on individual proficiency, vigilance, and self-management behaviors to achieve clinical benefits^7^. Therefore, constant evaluation and monitoring are essential to ensure good glycemic and health outcomes.

There is extensive scientific literature^8^ ^-^ ^10^ on diabetes mellitus (DM), and many studies relate it to self-care. However, to date, no attempt has been made to analyze self-care in people with DM1 using CSII.

Considering the relevance and specificities of care for people with chronic diseases, especially those with type 1 diabetes using CSII, the importance of research on self-care practices and their intervening factors, as well as ways to encourage these practices and offer qualified and effective care. This study aims to map the evidence available in the literature on self-care for people with DM1 using Continuous Insulin Infusion Systems.

## Method

### Protocol and registration

Scope review conducted based on the Manual for Evidence Synthesis, proposed by the Joanna Briggs Institute (JBI)^11^, according to the following steps: (1) research question, (2) eligibility criteria, (3) search strategy, (4) data extraction, (5) synthesis and presentation of main findings. The authors followed the recommendations of the Preferred Reporting Items for Systematic Reviews and Meta-Analyses - Extension for Scoping Reviews (PRISMA-ScR) checklist^12^.

This is a recommendation for a literature review used to map key concepts, summarize evidence, expand the breadth of the literature, and inform future research by drafting the review, developing inclusion criteria, search strategy, extraction, presentation, and summary of results and their implications for research and practice^11^.

This type of review was adopted to explore available evidence on self-care among people with DM1 using CSI, enabling an assessment of this attribute. The review protocol was registered in the Open Science Framework (OSF) under DOI: 10.17605/OSF.IO/A3U9E.

The PCC (Population, Concept, and Context) strategy was used to construct the research question, with the population (P) being people with type 1 diabetes mellitus, the concept (C) being self-care, and the context (C) being the use of the CSII, resulting in the following main guiding question: What is the scientific evidence on self-care among people with type 1 diabetes mellitus using the Continuous Insulin Infusion System?

### Eligibility criteria

The eligibility criteria were defined according to the PCC strategy: (1) Population (P), people diagnosed with DM1, of any age, of both sexes, with or without comorbidities; (2) Concept (C), studies focusing on self-care activities for people with DM1 using CSII; studies describing the process of evaluating these activities; and studies evaluating two or more self-care activities; and (3) Context (C), studies on the use of any brand/model of CSII.

Regarding the type of study, primary and secondary studies were selected, which could be quantitative, qualitative, or mixed, of any design or methodology, that considered self-care performed by people with DM1 using CSII. In addition, studies published in full, in any language, and in any format (national or international), without time restrictions, were considered.

Studies that included, in addition to people with T1DM using CSII, other participants, such as health professionals or family members, would be considered only for data on people with DM1 using CSII.

Studies involving individuals with type 2 diabetes mellitus or gestational diabetes using CSII, research protocols, opinion articles, editorials, letters to the editor, and abstracts in event proceedings were excluded, as they do not provide concise results on the self-care activities of individuals with DM1 using CSII.

### Sources of information

The search was conducted on October 2, 2024, using institutional access to the CAPES/*Acesso CAFe* (Academic Community) journal portal, when free access was not available. Thus, the search was conducted in the following databases/portals: Scientific Electronic Library Online (SciELO), PubMed, Scopus (Elsevier), Medical Literature Analysis and Retrieval System Online (MEDLINE) via BVS, Web of Science, ScienceDirect, Cumulative Index to Nursing and Allied Health Literature (CINAHL), Latin American and Caribbean Health Sciences Literature (LILACS) via BVS, Cochrane Library, and Embase.

The gray literature was retrieved from four sources: Google Scholar, the Brazilian Digital Library of Theses and Dissertations (BDBTD), the Thesis and Dissertation Catalog (CTD) of the Coordination for the Improvement of Higher Education Personnel (CAPES), and Open Access Theses and Dissertations (OATD). The documents were retrieved from the first ten pages of Google Scholar, without any time filter.

### Search

The search strategy was constructed using the Health Sciences Descriptors (DeCS), Medical Subject Headings (MeSH), EMTREE, and natural language, with the Boolean operators AND and OR. It was guided by the ECUS^13^ (Extraction, Conversion, Combination, Construction, and Usage) strategy, which presents a step-by-step guide for formulating a sensitive search strategy, consisting of the following stages: extraction, conversion, combination, construction, and usage (Figure 1).

**Figure 1:** ECUS* strategy for formulating the search strategy. Fortaleza, CE, Brazil, 2025

Stage	ECUS Strategy*		
	P^†^	C^‡^	C^§^
Extraction	People with type 1 diabetes mellitus	Self-Care	Insulin Infusion Systems
Conversion	Type 1 diabetes mellitus	Self-Care	Insulin Infusion Systems
Combination	Diabetes mellitus tipo 1	Self-Care; Self-Care; Self management	Insulin Infusion Systems; Insulin Pump
Construction	“Diabetes Mellitus Type 1” OR “Diabetes Mellitus, Type 1” OR “Diabetes, Type 1” OR “IDDM^||^” OR “Type 1 Diabetes” OR “Type 1 Diabetes Mellitus”	Self Care” OR “Self-Care”OR “Self-management”	“Insulin infusion systems” OR “Insulin pump”
Usage	“Diabetes Mellitus Type 1” OR “Diabetes Mellitus, Type 1” OR “Diabetes, Type 1” OR “IDDM” OR “Type 1 Diabetes” OR “Type 1 Diabetes Mellitus” AND “self care” OR “Self-care” OR “Self-management” AND “insulin infusion systems” OR “Insulin pump”		

*ECUS = Extraction, Conversion, Combination, Construction, and Use; ^†^P = Population; ^‡^C = Concept; ^§^C = Context; ^||^IDDM = Insulin-Dependent Diabetes Mellitus

With the definition established and tested, the terms and keywords were adapted to each database, using as a model the search strategy developed for the PubMed/MEDLINE: “Diabetes Mellitus type 1” OR “Diabetes Mellitus, Type 1” OR “Diabetes, Type 1” OR “IDDM” OR “Type 1 Diabetes” OR “Type 1 Diabetes Mellitus” AND “Self Care” OR “Self-care” OR “Self-management” AND “Insulin Infusion Systems” OR “Insulin Pump”.

### Selection of evidence sources

The studies obtained from the databases were imported into the free web version of the Rayyan^®^ reference manager, enabling automatic and manual analysis of duplicates. With the help of this tool, two researchers were able to blindly and independently evaluate two stages: (1) reading the title and abstract and (2) reading the full text. The selection and screening of studies were performed independently by two researchers, RRCB and CCS. Any disagreements would be resolved by a third reviewer (SKPO), who was not needed. According to the literature, conflicts can be resolved by consensus or by a third reviewer^14^.

After removing duplicates, articles were selected by reading their titles and abstracts in accordance with the study’s pre-established criteria. Thus, the studies were read in full to verify their permanence.

Data from the included articles were extracted using a Microsoft Excel^®^ spreadsheet that contained specific information on title, authors, year, country of origin, methodological approach, study objective, study population, number of participants, interventions, main results, and references. 

### Summary of results

After the data were extracted, thematic-categorical content analysis was used to identify the core meanings of that communication, whose frequency or presence was significant to the study objective^15^.

### Ethical aspects

As this is a scoping review study, approval by the Research Ethics Committee is not required.

## Results

### Selection of evidence sources

**Figure 2:**
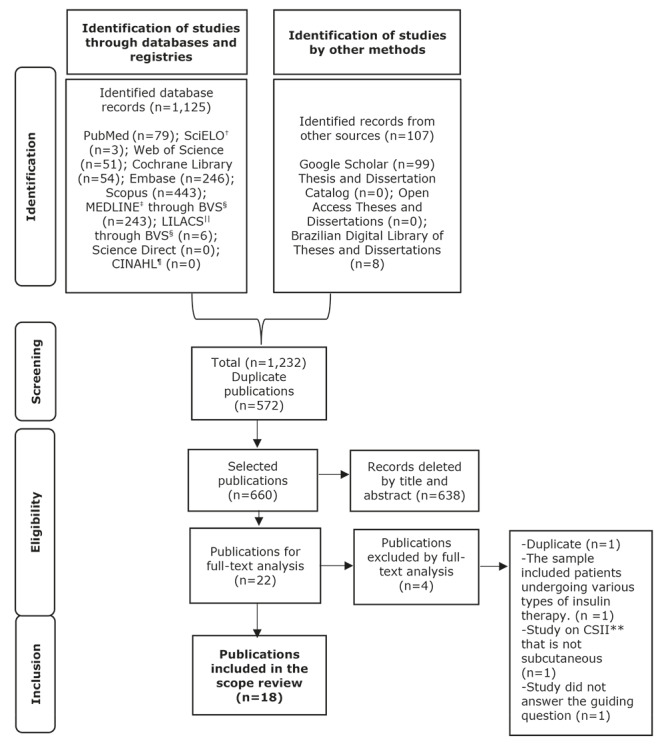
Flowchart of the study selection process adapted from Preferred Reporting Items for Systematic Review and Meta-Analyses extension for Scoping Reviews (PRISMA-ScR*)^12^

The search initially yielded 1,125 publications in databases and 107 in the gray literature, for a total of 1,232. Five hundred seventy-two were excluded because they were duplicates, leaving 660 for title and abstract reading. After the first analysis, with the reading of selected titles and abstracts, 638 were excluded, and 22 were selected for full reading. Of these, three were excluded because they did not address the research question, and one was duplicated. The final sample consisted of 18 articles (Figure 2).

*PRISMA-ScR = Preferred Reporting Items for Systematic reviews and Meta-Analyses extension for Scoping Reviews; ^†^SciELO = Scientific Electronic Library Online; ^‡^MEDLINE = Medical Literature Analysis and Retrieval System Online; ^§^VHL = Virtual Health Library; ^||^LILACS = Latin American and Caribbean Health Sciences Literature; ^¶^CINAHL = Cumulative Index to Nursing and Allied Health Literature; **CSII = Continuous Subcutaneous Insulin Infusion

### Characteristics of evidence sources

Figure 3 shows the characterization of the articles that were included as the final sample of this exploratory review. The publications span 2006 to 2023, with a significant increase in the past 10 years: 14 studies (73.6%) were published during this period.

In terms of language, the articles were published in English and Portuguese (n=18)^16^ ^-^ ^33^. The country with the most publications on the subject was the United States (n=10)^18^ ^,^ ^20^ ^,^ ^22^ ^,^ ^24^ ^,^ ^26^ ^-^ ^28^ ^,^ ^31^ ^-^ ^33^, followed by Sweden^21^ ^-^ ^29^ and England^23^ ^-^ ^30^ with two articles each, and then Saudi Arabia^16^, Brazil^17^, Denmark^19^, and Spain^25^ with one publication each (Figure 3).

Regarding the type of study, most of them were classified as quantitative^18^ ^-^ ^20^ ^,^ ^24^ ^-^ ^25^ ^,^ ^30^, followed by qualitative (n=9)^17^ ^,^ ^21^ ^-^ ^23^ ^,^ ^26^ ^,^ ^29^ ^,^ ^31^ ^-^ ^33^ and mixed-methods (n=6)^18^ ^-^ ^20^ ^,^ ^24^ ^-^ ^25^ ^,^ ^30^. Eleven studies were conducted with adults using CSII^16^ ^-^ ^17^ ^,^ ^19^ ^-^ ^21^ ^,^ ^25^ ^-^ ^28^ ^,^ ^30^ ^,^ ^32^; three studies with adolescents and parents^22^ ^,^ ^24^ ^,^ ^31^, one study with adolescents using CSII, parents, and pediatric diabetes nurses^29^, one with parents of children and adolescents using CSII and diabetes doctors^33^, and one study with adults using CSII and 20 specialist health professionals^23^. It is important to point out that only data referring to people with T1DM using CSII were used.

Results related to self-care among people with DM1 using CSII were identified in the selected articles; therefore, to describe them systematically, a content analysis was performed, identifying the most frequently cited characteristics through categorization.

**Figure 3:** Characterization of studies included in the scoping review, according to author/reference, year, title, methodological approach/study design, and study population. Fortaleza, CE, Brazil, 2025

Articles	Author/Year	Title	Methodological approach/study design	Study population
A*1	Al Hayek, 2021^16^	Effectiveness of the freestyle libre 2 flash glucose monitoring system on diabetes-self-management practices and glycemic parameters among patients with type 1 diabetes using insulin pump	Quantitative/ prospective cohort	Adults with T1DM^†^ using CSII^‡^
A*2	Brandão, et al., 2023^17^	*Aplicativos para autogestão do diabetes tipo 1 em usuários de sistema de infusão contínua de insulina*	Qualitative/ Integrative review	CSII^‡^ users with T1DM^†^
A*3	Faulds, et al., 2021^18^	Expect the unexpected: Adolescent and pre-teens’ experience of diabetes technology self-management	Mixed	Adolescents with T1DM† using CSII‡
A*4	Rytter, et al., 2023^19^	Associations between insulin pump self-management and HbA1c in type 1 diabetes	Mixed/Observational Study	Adults with T1DM^†^ using CSII^‡^
A*5	Faulds, et al., 2022^20^	Simulation platform development for diabetes and technology self-management	Mixed/Observational Study	Adults with T1DM^†^ using CSII^‡^
A*6	Persson, et al., 2022^21^	‘Striving for freedom or remaining with what is well-known’: a focus-group study of self-management among people with type 1 diabetes who have suboptimal glycemic control despite continuous subcutaneous insulin infusion	Qualitative	Adults with T1DM^†^ using CSII^‡^
A*7	Faulds, et al., 2021^22^	State of the science: A scoping review and gap analysis of adolescent insulin pump self-management	Qualitative	Adolescents with T1DM^†^ using CSII^‡^
A*8	Reidy, Foster, Rogers, 2020^23^	A facilitated web-based self-management tool for people with type 1 diabetes using an insulin pump: intervention development using the behavior change wheel and theoretical domains framework	Qualitative	Adultos com DM1^†^ em uso do SICI^‡^ e profissionais de saúde especialistas
A*9	O’Donnell, et al., 2021^24^	Pump It Up! A randomized clinical trial to optimize insulin pump self-management behaviors in adolescents with type 1 diabetes	Mixed/ Non-blind randomized clinical trial	Adolescents with DM1^†^ using CSII^‡^ and their parents
A*10	Quiró, et al., 2019^25^	Experiences and real-life management of insulin pump therapy in adults with type 1 diabetes	Mixed/Cross-sectional study	Adults with T1DM^†^ using CSII^‡^
A*11	Grando et al., 2017^26^	Characterization of Exercise and Alcohol Self-Management Behaviors of Type 1 Diabetes Patients on Insulin Pump Therapy	Qualitative	Adults with T1DM^†^ using CSII^‡^
A*12	Groat, et al., 2017^27^	Self-Management Behaviors in Adults on Insulin Pump Therapy	Quantity/Cohort	Adults with T1DM^†^ using CSII^‡^
A*13	Martyn-Nemeth, et al., 2017^28^	Fear of hypoglycemia: Influence on glycemic variability and self-management behavior in young adults with type 1 diabetes	Quantitative/Prospective Study	Adults with T1DM^†^ using CSII^‡^
A*14	Olinder, et al., 2011^29^	Clarifying responsibility for self-management of diabetes in adolescents using insulin pumps--a qualitative study	Qualitative	Adolescents with T1DM^†^ using CSII^‡^, parents, and pediatric diabetes nurse
A*15	Wilson, 2008^30^	Barriers to effective communication between patients using insulin pump therapy technology to enable intensive diabetes self-management and the health professionals providing their diabetes care	Mixed	Adults with T1DM^†^ using CSII^‡^
A*16	Berlin, et al., 2006^31^	Contextual Assessment of Problematic Situations Identified by Insulin Pump Using Adolescents and Their Parents	Qualitative	Adolescents with DM1^†^ using CSII^‡^ and their parents
A*17	Ritholz, et al., 2007^32^	Perceptions of Psychosocial Factors and the Insulin Pump	Qualitative	Adults with T1DM^†^ using CSII^‡^
A*18	Weissberg-Benchell, et al., 2017^33^	The use of Continuous Subcutaneous Insulin Infusion (CSII): parental and professional perceptions of self-care mastery and autonomy in children and adolescents	Qualitative	Parents of children and adolescents with T1DM^†^ using CSII^‡^ and diabetes physicians

*A = Article ; ^†^DM1 = Type 1 diabetes mellitus; ^‡^CSII = Continuous Subcutaneous Insulin Infusion System

### Summary of results

**Figure 4:**
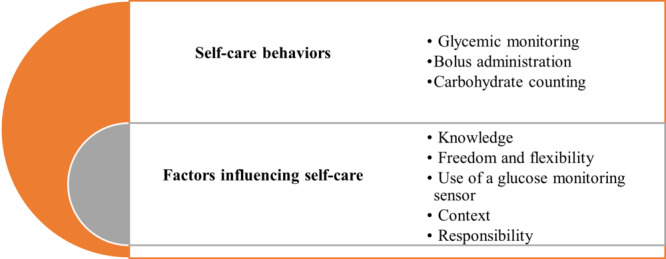
Classification of self-care among people with T1DM* using CSII^†^. Fortaleza, CE, Brazil, 2025

The results were divided into two categories: 1) Self-care behaviors and 2) Factors influencing self-care. Three self-care behaviors were listed: Glycemic management, Bolus administration, and Carbohydrate counting; and five factors influencing self-care: Knowledge; Freedom and flexibility; Use of glucose monitoring sensor; Context; and Responsibility (Figure 4).

*T1DM = Type 1 Diabetes Mellitus; ^†^CSII = Continuous Subcutaneous Insulin Infusion

### Self-care behaviors

#### Glycemic monitoring

Increased frequency of self-monitoring of capillary blood glucose (SMBG) was associated with lower glycated hemoglobin (HbA1c) in people with DM1 using CSII^22^ ^,^ ^25^ ^,^ ^33^. In addition to the frequency of SMBG, adolescents who recorded a glucose reading before each meal had significantly lower HbA1c than those who did not^22^.

#### Bolus administration

Increasing the frequency of CSII insulin boluses was associated with a decrease in HbA1c in people with DM1^22^ ^,^ ^25^ ^,^ ^33^. Increasing the number of daily insulin boluses had the greatest impact on the number of capillary glucose readings within the target range on that day (r = 0.93)^27^.

Studies have examined the timing and frequency of insulin bolus administration^22^ ^,^ ^24^ ^-^ ^25^ ^,^ ^27^. It was found that the nighttime bolus had the most significant effect on HbA1c, with the administration of all nighttime boluses associated with a reduction in HbA1c of 43.2 (21.6-64.7) mmol/mol [5.59% (95% CI: 2.79%, 8.37%)] compared to children who did not take boluses at nighttime meals^22^.

In this regard, some authors^25^ ^-^ ^33^ reported that the bolus calculator has been frequently used among people using CSII. A study^33^ showed that 79% of patients using CSII used the calculator daily. Considering the total number of bolus/day, another study found that most people using CSII used the bolus calculator rather than manual boluses (20.9%)^25^.

The use of insulin bolus calculators has been associated with better HbA1c levels. A study^22^ conducted in the US found that adolescents who always used their insulin pump’s bolus calculator had significantly lower HbA1c levels and were more likely to achieve an HbA1c target of ≤7.5%, compared to adolescents who used the bolus calculator less than 50% of the time (7.8 ± 0.2% vs. 8.5%, p = 0.015; 67.9 ± 19.4% vs. 30.8 ± 7.1%, p = 0.0731, respectively).

#### Carbohydrate counting

Carbohydrate counting was observed during the evaluation of self-care behaviors in people with T1DM using the CSII^19^ ^,^ ^24^ ^-^ ^27^.

It was noted that patients using CSII had a high frequency of carbohydrate intake (3 or more times/day), with an average of 76.6%. This demonstrates that using CSII enables individuals to control their daily carbohydrate intake, thereby promoting greater effectiveness in this behavior. Perhaps this is because the amount of carbohydrates ingested must be entered into the bolus calculator^27^.

Adherence levels to carbohydrate counting were similar among those using continuous glucose monitoring (29.7%) and capillary blood glucose monitoring (33.3%). Five participants demonstrated adherence to this behavior 100% of the time, while only one participant showed a maximum of two carbohydrate entries per day. Carbohydrates were documented an average of 3.9 times per day^27^.

The total carbohydrate intake per day was higher (141 ± 58 *vs*. 121 ± 64 g/day; p < 0.05) among participants using CSII who had HbA1c ≤7.5%^25^. This data demonstrates that the more frequently carbohydrate records are entered into the device, the better the patient’s glycemic control tends to be.

### Factors influencing self-care

#### Knowledge

Patients and healthcare professionals must be knowledgeable about CSII therapy to use it effectively and administer insulin as needed. The studies analyzed addressed knowledge and self-care in two dimensions: knowledge of the person using the CSII or the healthcare professional^20^ ^-^ ^22^ and the need for knowledge support^23^ ^-^ ^30^.

Adolescents who use CSII and have greater knowledge about diabetes tend to report greater independence in self-managing the disease compared to those with more limited knowledge^22^. A deeper understanding of the condition and its treatment facilitates more effective and safer self-management.

In this context, a study^20^ conducted with CSII users, using a realistic simulation, identified self-management errors during the simulation. Among them, the choice of carbohydrate-free foods to treat or prevent hypoglycemia, failure to check ketones, inability to change the pump infusion site, and failure to avoid hypoglycemia by consuming carbohydrates or temporarily reducing the basal rate before exercise stood out.

The lack of information about CSII treatment and the absence of treatment support were evident in the studies analyzed^23^ ^-^ ^30^. CSII users interviewed in one of the studies reported not receiving support from health professionals for intensive diabetes self-management or for CSII use^30^.

In the same study^30^, CSII users reported that their diabetes treatment was not patient-centered, with 88.1% of participants reporting inadequate explanations from health professionals. In addition, a large proportion (91.0%) said that their diabetes care and support needs were not met due to a lack of professional healthcare training in the use of CSII therapy.

Person-centered diabetes education can improve HbA1C levels, and personalized education can meet the expectations of people with DM1 who need individualized support^21^.

#### Freedom and flexibility

Participants appreciated the freedom and flexibility provided by CSII treatment^21^ ^-^ ^32^. After the initial struggles to start treatment, they felt they had the freedom to live their lives as they wished and often adjusted their therapy to their current situation^21^.

People chose a basic schedule for daily life but often switched to other programs depending on the day’s activities, such as bolus doses were frequently used according to meals, and the use of sensors for better treatment adjustments. In addition, they reported that the use of the device led to an improvement in HbA1C from previous levels^21^.

When analyzing the self-care of people using CSII, a study conducted with groups of patients with low, medium, and high HbA1c, identified that all groups discussed the insulin pump as making self-care more “convenient”, expressed in terms of having greater “flexibility” and “freedom”^32^.

Members of the low, medium, and high HbA1c groups reported being actively involved in their diabetes self-care. They stated that, in addition to “convenience”, the real benefit of the pump was improved glycemic control. Participants in the high A1C group, on the other hand, focused mainly on the “convenience” of CSII and expressed reluctance to perform expected self-care behaviors, such as not valuing food records or blood glucose logs, and admitted to sometimes forgetting to use bolus insulin^32^.

#### Responsibility

Studies^29^ ^-^ ^33^ addressing the issue of diabetes responsibility and self-management were conducted with adolescents and their parents. One of the studies^29^ pointed out that lack of responsibility appears to be the main reason for missed bolus doses and insufficient self-management.

Responsibility can be explained in three subcategories: Distribution of responsibility, Transfer of responsibility, and Clarification of responsibility^29^.

Responsibility for diabetes self-management is clearly distributed among those who use most of their doses as boluses. Some of these adolescents said that their parents did not know much about CSII treatment and therefore did not want their help. They wanted to take care of themselves. Since CSII treatment is more technological than injections, many adolescents handle pumps more easily than their parents. This causes parents to relinquish their responsibility, leaving them to perform their self-care^29^.

The optimal transfer of responsibility is when it gradually shifts from parents to adolescents. They may need more support when they are sick or when their blood glucose is too high or too low, making it difficult to control. Sometimes, adolescents can take on more responsibility for a while, doing more things on their own without losing control of their diabetes^29^.

Adolescents and parents need to negotiate and clarify who is responsible for diabetes self-management. This negotiation needs to be ongoing, and diabetes care teams can support these negotiations. Parents need to know what they can do to facilitate adolescents’ self-management^29^.

#### Use of the glucose monitoring sensor

The flash glucose monitoring (FGM) system provides patients with an easy way to continuously monitor interstitial fluid glucose, enabling more active self-care^16^.

A study^16^ conducted with patients using the CSII found that after 12 weeks of using FGM, the self-care activities that showed a greater degree of change were: 1) Glucose management: the items “check my blood sugar levels carefully and attentively” (p=0.041) and “record blood sugar levels regularly” (p=0.032); 2) Dietary control: the items “The foods I choose to eat make it easier to achieve optimal blood sugar levels” (p=0.024) and “I occasionally eat too many sweets or other foods rich in carbohydrates” (p=0.049); 3) Healthcare use: the items “I tend to avoid medical appointments related to diabetes” (p=0.031) and “My self-care with diabetes is poor” (p=0.021).

Additionally, patients with a higher number of daily FGM tests showed significantly improved HbA1c levels^16^.

### Context

One of the studies^31^, conducted with adolescents using CSII and their parents, identified self-management as an area that, according to them, presented difficulties. However, a different picture of self-management emerged when the context was considered. Both mothers and fathers reported more problems with self-management across family and multiple contexts, whereas young people reported more frequent problems with self-management in a peer context. 

When considering the general grouping of the most frequently reported situations, different patterns emerge for each family member. For young people, the modal interaction of content and context was complex with self-management within a social context, while for mothers, it was self-management within a family context. Fathers, on the other hand, reported two modal content domains (hypoglycemia and pump malfunction), both occurring more frequently within a family context^31^.

## Discussion

Self-care in patients with DM1 using CSII is essential to ensure effective glycemic control, reduce acute and chronic complications, and promote quality of life. CSII offers significant benefits but requires the patient to commit and have knowledge for its safe and effective use.

In this sense, glycemic monitoring is key to the success of CSII treatment, which, in terms of self-care, involves interpreting data and adjusting insulin doses when necessary (meals, exercise, corrections).

For proper glycemic management in people with DM1, continuous insulin therapy, self-monitoring of blood glucose, follow-up with qualified professionals for support and health education, and a healthy lifestyle are necessary^34^.

Ideally, self-monitoring should be performed seven to nine times a day, according to the therapy and need, once on an empty stomach upon waking up, every time before meals (pre-prandial), 2 hours after meals (postprandial), and at bedtime^35^.

The healthcare team and the patient must be trained and committed to addressing the causes and effects identified through self-monitoring. When patients are involved in this self-care behavior, the likelihood of clinical improvement is even greater, as it is an effective motivational tool for them^35^.

Living with DM1 is a challenging experience that generates conflicts and difficulties in the face of the unpredictability of the disease, due to the demands and lifestyle changes required by treatment. From the moment of diagnosis, care can become complex, involving various daily tasks that affect family dynamics and pose obstacles to treatment adherence, and is influenced by clinical and sociodemographic factors that can impact health status^36^. Therefore, health education by a multidisciplinary health team trained to welcome this family and this patient is of utmost importance.

In this context, it is essential to provide healthcare professionals with technological resources so they can act as facilitators in care and education processes, helping patients with DM1 reevaluate their behaviors and thoughts to promote more effective healthcare^37^.

In CSII treatment, exogenous insulin replacement can be performed in different ways, such as basal insulin, pre-meal bolus insulin, and insulin to correct pre- and post-prandial hyperglycemia or between meals^38^.

In some CSII models, different types of boluses can be administered immediately or over a more extended period, depending on the patient’s needs at the time^39^. On the one hand, insulin is released continuously and programmed (basal rate) to control hepatic glucose production during the night and late postprandial period (> 4 hours). On the other hand, it is released on demand (bolus) before meals (prandial bolus) or to correct hyperglycemia (correction bolus)^38^.

Most CSII models feature a bolus calculator that performs bolus infusion calculations. The patient only needs to enter their blood glucose level and whether they will be ingesting carbohydrates (CH), and the amount in grams or servings (1 serving = 10 g of CH)^40^. This facilitates insulin administration, as the person does not need to perform any calculations.

Some food management apps have an option for automatic insulin dose calculation. It is still a challenge for many people with DM1 to estimate the appropriate insulin dose. The difficulty of calculating adequate insulin doses may be why many people do not achieve their therapeutic goals, as evidenced by high glycated hemoglobin levels^41^.

Implementing a diet to maintain nutritional parameters within the desired range has proven to be one of the most significant challenges in treating people with diabetes. To create a personalized meal plan, one of the main approaches to be implemented for people with DM1 is carbohydrate counting. This is based on the total amount of carbohydrates a person can or should consume per day, considering their routine, schedule, and medications^42^.

A Brazilian study^43^ evaluated predictive factors for glycemic control in children and adolescents with DM1, and the results indicated that disease duration and insulin dose were directly associated with higher HbA1c levels, while carbohydrate counting was associated with a reduction in HbA1c.

Many people with DM1 may be candidates for CSII use. However, for the treatment to be successful, they must be motivated, aware, and possess technical knowledge and self-management skills. In addition, they must receive adequate training and be continuously monitored to ensure the correct use of the system^44^.

Regarding knowledge of CSII, the studies in this review found that the greater the understanding of patients and professionals, the better the management and safety of the treatment.

Adverse events can be detected and prevented using problem-solving strategies, highlighting the importance of patient education when starting CSII therapy^45^. In addition, some errors and adverse events can occur due to a lack of knowledge.

In this context, it is intuitive to consider that effective control of DM1 depends not only on the proper use of medications, but also on patients’ knowledge about their treatment, the adoption of healthy eating habits, exercise, and self-monitoring of blood glucose^46^.

CSII has proven to be an effective method of glycemic management, providing greater convenience for users and greater freedom in their daily tasks. It is associated with improved HbA1c levels and a lower rate of nocturnal hypoglycemia. However, the person needs to be motivated to learn about the methods available in CSII to achieve glycemic control^47^.

With increased responsibilities following a diagnosis of DM1, young people need to manage their self-care together with their guardians, which results in greater responsibility from an early age in the face of daily challenges. These individuals must receive support, especially from their families, to avoid emotional overload that could compromise their self-care^48^.

The acquisition of self-care responsibility by adolescents is usually supported by ongoing parental support and begins with interdependence in diabetes management tasks. There is a natural transition in care responsibilities as adolescents move away from their families and spend more time on academic, sports, social, and even work-related activities^48^.

Systematic reviews^49^ ^-^ ^50^ found that continuous glucose monitoring (CGM) was easy to use and improved patient empowerment and autonomy in managing glucose levels, thereby preventing hypoglycemia.

Corroborating these findings, several studies^51^ ^-^ ^53^ reported that the frequency of self-monitoring is associated with improved HbA1c levels and reduced diabetes-related complications, suggesting a direct association between daily monitoring and diabetes control.

The results of this study indicate that self-care for individuals with DM1 using CSII is essential, as it involves behaviors and factors that influence self-care, thereby improving patients’ quality of life and minimizing health complications.

Despite this review’s significant contributions to the field and practice of science, it has limitations. Although it is a robust methodology for synthesizing health evidence, this scope review, unlike the traditional systematic review, does not incorporate an assessment of methodological quality or a combined effect estimate for interventions. Consequently, the studies included in this work were not evaluated and synthesized considering their level of evidence.

Regarding the identified gaps, there is limited evidence on self-care among people with DM1 using CSSII. Therefore, these issues highlight the urgent need for additional research, as well as interdisciplinary collaboration among health professionals, to assess self-care in this specific population across different age groups and to better understand this phenomenon and their needs at various stages of life.

## Conclusion

In summary, this review provided a comprehensive overview of self-care in people with type 1 diabetes using CSII, highlighting the behaviors and factors that influence self-care. Given this, it is essential to understand self-care in the management of DM1 with CSII, given its complexity and the ongoing demands of treatment.

It is worth noting that in-depth knowledge about self-care allows professionals to update and make better decisions regarding individualized treatment plans; and will enable people with DM to seek information and actions on daily care for the proper management of diabetes, especially the safe handling of CSII. The need for future intervention studies that optimize self-care in this population is highlighted.

## Data Availability

All data generated or analysed during this study are included in this published article.
