# Supplementary material for: Continuous Insulin Infusion System and self-care in Type 1 Diabetes Mellitus: scope review
Source: Rev Lat Am Enfermagem. 2026 Jun 15;34:e4852. doi: 10.1590/1518-8345.8001.4852 (PMC13277350; doi:10.1590/1518-8345.8001.4852)
Supplement: Translation

# Translation: Sistema de Infusão Contínua de Insulina e autocuidado no Diabetes Mellitus Tipo 1: revisão de escopo

**DOI:** 10.1590/1518-8345.8001.4853

**Authors:** Raquel Rodrigues da Costa Brilhante, Carla Cristina de Sordi, Paulo Weslen Carneiro Gonçalves, Vanessa de Araujo Lima Freire, Amelina de Brito Belchior, Maria Luiza Pereira Costa, Raquel Sampaio Florêncio, Sherida Karanini Paz de Oliveira

**Affiliations:** 1 Universidade Estadual do Ceará, Fortaleza, CE, Brasil.

**Keywords:** Insulina, Sistemas de Infusão de Insulina, Autocuidado, Autogestão, Conhecimento, Diabetes Mellitus

## Abstract

**Objetivo:**

mapear as evidências disponíveis na literatura sobre o autocuidado de pessoas com Diabetes Mellitus Tipo 1 em uso do Sistema de Infusão Contínua de Insulina.

**Método:**

trata-se de uma revisão de escopo baseada nos princípios preconizados pelo JBI e nas diretrizes do PRISMA para a extração dos dados, realizada em dez bases de dados e da literatura cinzenta. Utilizou-se a estratégia PCC para a coleta de dados. A seleção dos estudos foi realizada após a remoção de duplicatas, avaliação individual e em pares. Para apresentação dos resultados, utilizou-se análise descritiva e diagrama.

**Resultados:**

uma análise dos 18 artigos selecionados mostrou que existem três comportamentos de autocuidado: manejo glicêmico, *bolus* de insulina e contagem de carboidratos. Os fatores que influenciam esses comportamentos foram o Conhecimento, Liberdade/flexibilidade, Uso do sensor, Contexto e Responsabilidade.

**Conclusão:**

os estudos evidenciam que um conhecimento aprofundado sobre o autocuidado permite aos profissionais tomar melhores decisões quanto ao plano terapêutico individualizado; e as pessoas em uso do Sistema de Infusão Contínua de Insulina, buscar ações e informações em relação aos cuidados diários, para o adequado manejo do diabetes. Destaca-se a necessidade de estudos futuros de intervenção que otimizem o autocuidado nessa população.

## Destaques

**(1)** Identifica os comportamentos de autocuidado de pessoas com DM1. **(2)** Analisa os fatores que influenciam os comportamentos de autocuidado. **(3)** Ajuda os profissionais a tomar decisões quanto ao plano terapêutico individualizado. **(4)** Auxilia profissionais a buscar ações e informações em relação aos cuidados diários.

## Introdução

O Diabetes Mellitus Tipo 1 (DM1) é uma condição autoimune caracterizada pela destruição das células beta do pâncreas, geralmente levando à deficiência absoluta de insulina^1^. Embora seja o tipo mais comum de diabetes mellitus em crianças e adolescentes, estudiosos indicam que há mais casos novos de DM1 diagnosticados na vida adulta do que na infância e adolescência^2^.

Em 2022, estimava-se que havia 8,75 milhões de indivíduos com DM1 em todo o mundo. O Brasil encontrava-se entre os dez países com o maior número de casos prevalentes de DM1 em todas as idades^3^. Esse cenário epidemiológico exige estratégias que estimulem as práticas de autocuidado para alcançar resultados terapêuticos satisfatórios e prevenir complicações. As práticas de autocuidado incluem planejamento alimentar, atividade física, uso regular de medicamentos, em especial a insulina, verificação da glicemia capilar de rotina^4^.

Para um manejo glicêmico satisfatório do DM1, enfatiza-se um tratamento intensivo de insulinoterapia que deve mimetizar a secreção fisiológica de insulina^5^. Dentre esses tratamentos, o Sistema de Infusão Contínua de Insulina (SICI) é um dispositivo eletrônico que infunde insulina através do tecido subcutâneo de forma ininterrupta, similar ao que ocorre no organismo da pessoa sem a doença, para tentar obter euglicemia entre as refeições e libera insulina nos horários de alimentação. O uso do SICI pode proporcionar maior segurança e autonomia em relação ao tratamento do DM1^6^.

Para uso da SICI, a pessoa com a doença precisa adquirir e desenvolver conhecimentos e habilidades de cuidados apropriados com o tratamento. Para tanto, faz-se necessária uma abordagem interprofissional com foco na educação em diabetes para o cuidado do paciente. Dispositivos para diabetes, como o SICI, são máquinas complexas que dependem muito da proficiência individual, vigilância e comportamentos de autogestão para atingir benefícios clínicos^7^. Por isso, é importante sua avaliação e monitoramento constantes para garantir bons resultados glicêmicos e de saúde.

Existe uma extensa literatura científica^8^ ^-^ ^10^ sobre Diabetes Mellitus (DM) e muitos estudos relacionando-o ao autocuidado. No entanto, até o presente momento, nenhuma tentativa anterior de estabelecer uma análise do autocuidado de pessoas com DM1 em uso do SICI.

Ao considerar a relevância e as especificidades do cuidado às pessoas com doenças crônicas, em especial aquelas com DM1 em uso de SICI, a importância de investigações sobre práticas de autocuidados e seus fatores intervenientes, bem como formas de estimular essas práticas e oferecer um cuidado qualificado e efetivo. Assim, este estudo objetiva mapear as evidências disponíveis na literatura sobre o autocuidado de pessoas com DM1 em uso do SICI.

## Método

### Protocolo e registro

Revisão de escopo conduzida a partir do *Manual for Evidence Synthesis*, proposto pela *Joanna Briggs Institute* (JBI)^11^, conforme as etapas: (1) questão de pesquisa, (2) critérios de elegibilidade, (3) estratégia de busca, (4) extração dos dados, (5) síntese e apresentação dos principais achados. Os autores seguiram as recomendações do *checklist Preferred Reporting Items for Systematic and Meta-Analyses - Extension for Scoping Reviews* (PRISMA-ScR)^12^. 

Essa é uma recomendação de revisão de literatura utilizada para mapear os principais conceitos, resumir evidências, possibilitar maior amplitude da literatura e informar pesquisas futuras que perpassam a realização de um esboço da revisão, a elaboração de critérios de inclusão, estratégia de busca, extração, apresentação e resumo dos resultados e suas implicações para a pesquisa e para a prática^11^.

Adotou-se esse tipo de revisão para explorar as evidências disponíveis sobre autocuidado de pessoas com DM1 em uso de SICI, permitindo uma apreciação desse atributo. O protocolo de revisão foi registrado no *Open Science Framework* (OSF) sob o DOI: 10.17605/OSF.IO/A3U9E.

A estratégia PCC (População, Conceito e Contexto) foi utilizada para a construção da pergunta de pesquisa, sendo população (P) pessoas com DM1, o conceito (C) autocuidado e o contexto (C) uso do SICI, resultando na seguinte questão norteadora principal: Quais as evidências científicas sobre o autocuidado de pessoas com DM1 em uso do Sistema de Infusão Contínua de Insulina?

### Critérios de elegibilidade

Os critérios de elegibilidade foram definidos conforme a estratégia PCC: (1) População (P), pessoas com diagnóstico de DM1, de qualquer idade, de ambos os sexos, com ou sem comorbidades; (2) Conceito (C), estudos com abordagem sobre atividades de autocuidado para pessoas com DM1 em uso do SICI; estudos que descrevessem o processo de avaliação dessas atividades; e estudos que avaliassem duas ou mais atividades de autocuidado; e (3) Contexto (C), estudos sobre o uso de qualquer marca/modelo de SICI. 

Quanto ao tipo de estudo, elegeram-se estudos primários e secundários, podendo ser quantitativos, qualitativos ou mistos, de qualquer desenho ou metodologia, que considerassem o autocuidado realizado por pessoas com DM1 em uso do SICI. Além disso, consideraram-se estudos publicados na íntegra, em formato completo, nacionais ou internacionais, em qualquer idioma, sem recorte temporal.

Estudos que envolvessem, além das pessoas com DM1 em uso do SICI, outros participantes, como profissionais da saúde ou familiares, foram considerados apenas os dados referentes às pessoas com DM1 em uso do SICI. 

Foram excluídos estudos com pessoas com Diabetes Mellitus Tipo 2 ou Diabetes gestacional em uso do SICI, protocolos de pesquisa, artigos de opinião, editoriais, cartas ao editor e resumos em anais de eventos, ao passo que não dispõem de resultados concisos sobre as atividades de autocuidado de pessoas com DM1 em uso do SICI.

### Fontes de informação

A busca ocorreu no dia 02 de outubro de 2024, utilizando-se do acesso institucional do portal de periódicos da CAPES/Acesso CAFe (Comunidade Acadêmica), quando não disponível o acesso de forma livre e gratuita. Assim, foi realizada a busca nas seguintes bases de dados/portal: *Scientific Electronic Library Online* (SciELO), PubMed, Scopus (Elsevier), *Medical Literature Analysis and Retrieval System Online* (MEDLINE) via BVS, *Web of Science*, *ScienceDirect*, *Cumulative Index to Nursing and Allied Health Literature* (CINAHL), Literatura Latino-Americana e do Caribe em Ciências da Saúde (LILACS) via BVS, *Cochrane Library* e Embase. 

A literatura cinza foi recuperada em quatro fontes: *Google Scholar*, Biblioteca Digital Brasileira de Teses e Dissertações (BDBTD), Catálogo de Teses e Dissertações (CTD) da Coordenação de Aperfeiçoamento de Pessoal de Nível Superior (CAPES), *Open Access Theses and Dissertations* (OATD). A recuperação dos documentos no *Google Scholar* foi realizada nas dez primeiras páginas, sem filtro de período.

### Busca

A estratégia de busca foi construída com base nos Descritores em Ciências da Saúde (DeCS), *Medical Subject Headings* (MeSH), EMTREE e linguagem natural em conjunto com os operadores booleanos AND e OR. Foi direcionada pela estratégia ECUS^13^ (Extração, Conversão, Combinação, Construção e Uso), que apresenta o passo a passo para a formulação de uma estratégia de busca sensível cujas etapas são: extração, conversão, combinação, construção e uso (Figura 1).

**Figura 1:** Estratégia ECUS* para a formulação da estratégia de busca. Fortaleza, CE, Brasil, 2025

Etapas	Estratégia ECUS*		
	P^†^	C^‡^	C^§^
Extração	Pessoas com diabetes mellitus tipo 1	Autocuidado	Sistemas de Infusão de Insulina
Conversão	Diabetes mellitus tipo 1	Self Care	Insulin Infusion Systems
Combinação	Diabetes mellitus type 1	Self Care; Self-Care; Self management	Insulin Infusion Systems; Insulin Pump
Construção	“Diabetes Mellitus Type 1” OR “Diabetes Mellitus, Type 1” OR “Diabetes, Type 1” OR “IDDM^||^” OR “Type 1 Diabetes” OR “Type 1 Diabetes Mellitus”	Self Care” OR “Self-Care”OR “Self-management”	“Insulin infusion systems” OR “Insulin pump”
Uso	“Diabetes Mellitus Type 1” OR “Diabetes Mellitus, Type 1” OR “Diabetes, Type 1” OR “IDDM” OR “Type 1 Diabetes” OR “Type 1 Diabetes Mellitus” AND “self care” OR “Self-care” OR “Self-management” AND “insulin infusion systems” OR “Insulin pump”		

*ECUS = Extração, Conversão, Combinação, Construção e Uso; ^†^P = População; ^‡^C = Conceito; ^§^C = Contexto; ^||^IDDM = *Insulin-Dependent Diabetes Mellitus*

Com a definição realizada e testada, os termos e palavras-chave foram adaptados a cada base de dados, utilizando como modelo a estratégia de busca elaborada para a *PubMed*/Medline: “Diabetes Mellitus type 1” OR “Diabetes Mellitus, Type 1” OR “Diabetes, Type 1” OR “IDDM” OR “Type 1 Diabetes” OR “Type 1 Diabetes Mellitus” AND “Self Care” OR “Self-care” OR “Self-management” AND “Insulin Infusion Systems” OR “Insulin Pump”.

### Seleção de fontes de evidências

Os estudos obtidos nas bases foram importados para o gerenciador de referências *Rayyan* ^*®*^ versão *Web* gratuita, possibilitando a análise automática e manual de duplicatas. Com o auxílio dessa ferramenta, dois pesquisadores puderam, de forma cega e independente, realizar a avaliação em duas etapas: (1) leitura do título e resumo e (2) leitura do texto completo. A seleção e a triagem dos estudos foram realizadas por dois pesquisadores, RRCB e CCS, de forma independente. Eventuais discordâncias seriam resolvidas por meio de um terceiro revisor (SKPO), o qual não foi necessário acionar. Segundo a literatura, as discordâncias podem ser resolvidas por consenso ou por meio de um terceiro revisor^14^. 

Após remoção das duplicadas, procedeu-se à seleção dos artigos por meio da leitura dos títulos e resumos, com base nos critérios preestabelecidos no estudo. Assim, os estudos foram lidos na íntegra para verificação da permanência. 

Os dados dos artigos incluídos foram extraídos usando uma planilha própria do *software* Microsoft Excel^®^, contendo informações específicas sobre título, autores, ano, país de origem, abordagem metodológica, objetivo do estudo, população estudada, número de participantes, intervenções, principais resultados e referência. 

### Síntese dos resultados

Após os dados serem extraídos, foi utilizada a análise de conteúdo, do tipo temático-categorial, quando se descobrem os núcleos de sentido daquela comunicação cuja frequência ou presença signifique algo para o objetivo do estudo^15^.

### Aspectos éticos

Por se tratar de um estudo de revisão de escopo, é dispensada sua aprovação pelo Comitê de Ética em Pesquisa.

## Resultados

### Seleção de fontes de evidências

A pesquisa resultou inicialmente em 1125 publicações nas bases de dados e 107 na literatura cinzenta, totalizando 1232. Foram excluídas 572 por serem duplicadas, restando 660 para a leitura de título e resumo. Após a primeira análise, com a leitura de títulos e resumos triados, foram excluídos 638 e selecionados 22 para a leitura na íntegra. Desses, três foram excluídos, pois não respondiam à pergunta de pesquisa, e um estava duplicado. A amostra final foi composta por 18 artigos (Figura 2).

**Figura 2:** Fluxograma do processo de seleção dos estudos adaptado do *Preferred Reporting Items for Systematic Review and Meta-Analyses extension for Scoping Reviews* (PRISMA-ScR*)^12^

*PRISMA-ScR = *Preferred Reporting Items for Systematic reviews and Meta-Analyses extension for Scoping Reviews*; ^†^SciELO = *Scientific Electronic Library Online*; ^‡^MEDLINE = *Medical Literature Analysis and Retrieval System Online*; ^§^BVS = Biblioteca Virtual em Saúde; ^||^LILACS = Literatura Latino-Americana e do Caribe em Ciências da Saúde; ^¶^CINAHL = *Cumulative Index to Nursing and Allied Health Literature*; ^**^SICI = Sistema de Infusão Contínua de Insulina

### Características das fontes de evidências

Na Figura 3 é apresentada a caracterização dos artigos que foram incluídos como amostra final desta revisão de escopo. As publicações encontram-se entre 2006 e 2023, sendo observado aumento significativo das publicações nos últimos 10 anos, já que 14 estudos (73,6%) foram divulgados nesse período.

Em relação ao idioma, os artigos foram publicados em inglês e português (n=18)^16^ ^-^ ^33^. O país com mais publicações sobre o tema foi os Estados Unidos (n=10)^18^ ^,^ ^20^ ^,^ ^22^ ^,^ ^24^ ^,^ ^26^ ^-^ ^28^ ^,^ ^31^ ^-^ ^33^ seguido de Suécia^21^ ^-^ ^29^ e Inglaterra^23^ ^-^ ^30^ com dois artigos cada, e depois Arábia Saudita^16^, Brasil^17^, Dinamarca^19^ e Espanha^25^ com uma publicação cada (Figura 3). 

Sobre o tipo de estudo, grande parte deles definiram-se como quantitativos (n=3)^16^ ^,^ ^27^ ^-^ ^28^, seguidos de qualitativos (n=9)^17^ ^,^ ^21^ ^-^ ^23^ ^,^ ^26^ ^,^ ^29^ ^,^ ^31^ ^-^ ^33^ e mistos (n=6)^18^ ^-^ ^20^ ^,^ ^24^ ^-^ ^25^ ^,^ ^30^. Onze estudos foram realizados com adultos em uso do SICI^16^ ^-^ ^17^ ^,^ ^19^ ^-^ ^21^ ^,^ ^25^ ^-^ ^28^ ^,^ ^30^ ^,^ ^32^; três estudos com adolescentes e pais^22^ ^,^ ^24^ ^,^ ^31^, um estudo com adolescentes em uso do SICI, pais e enfermeira pediátrica de diabetes^29^, um com pais de crianças e adolescentes em uso do SICI e médicos de diabetes^33^ e um estudo com adultos em uso do SICI e 20 profissionais de saúde especialistas^23^. Vale ressaltar que somente os dados referentes às pessoas com DM1 em uso de SICI foram utilizados.

Os resultados relacionados ao autocuidado de pessoas com DM1 em uso de SICI foram identificados nos artigos selecionados; diante disso, para descrever de forma sistematizada, foi realizada uma análise do conteúdo abordado, identificando no texto as características mais citadas por meio da categorização.

**Figura 3:** Caracterização dos estudos incluídos na revisão de escopo, segundo autor/referência, ano, título, abordagem metodológica/desenho do estudo e população estudada. Fortaleza, CE, Brasil, 2025

Artigos	Autor/Ano	Título	Abordagem metodológica/desenho do estudo	População estudada
A*1	Al Hayek, 2021^16^	*Effectiveness of the freestyle libre 2 flash glucose monitoring system on diabetes-self-management practices and glycemic parameters among patients with type 1 diabetes using insulin pump*	Quantitativo/coorte prospectivo	Adultos com DM1^†^ em uso do SICI^‡^
A*2	Brandão, et al., 2023^17^	Aplicativos para autogestão do diabetes tipo 1 em usuários de sistema de infusão contínua de insulina	Qualitativo/Revisão integrativa	Usuários do SICI^‡^ com DM1^†^
A*3	Faulds, et al., 2021^18^	*Expect the unexpected: Adolescent and-pre-teens’ experience of diabetes technology self-management*	Misto	Adolescentes com DM1^†^ em uso do SICI^‡^
A*4	Rytter, et al., 2023^19^	*Associations between insulin pump self-management and HbA1c in type 1 diabetes*	Misto/Estudo Observacional	Adultos com DM1^†^ em uso do SICI^‡^
A*5	Faulds, et al., 2022^20^	*Simulation platform development for diabetes and technology self-management*	Misto/Estudo Observacional	Adultos com DM1^†^ em uso do SICI^‡^
A*6	Persson, et al., 2022^21^	*‘Striving for freedom or remaining with what is well-known’: a focus-group study of self-management among people with type 1 diabetes who have suboptimal glycaemic control despite continuous subcutaneous insulin infusion*	Qualitativo	Adultos com DM1^†^ em uso do SICI^‡^
A*7	Faulds, et al., 2021^22^	*State of the science: A scoping review and gap analysis of adolescent insulin pump self-management*	Qualitativo	Adolescentes com DM1^†^ em uso de SICI^‡^
A*8	Reidy, Foster, Rogers, 2020^23^	*A facilitated web-based self-management tool for people with type 1 diabetes using an insulin pump: intervention development using the behavior change wheel and theoretical domains framework*	Qualitativo	Adultos com DM1^†^ em uso do SICI^‡^ e profissionais de saúde especialistas
A*9	O’Donnell, et al., 2021^24^	*“Pump It Up! A randomized clinical trial to optimize insulin pump self-management behaviors in adolescents with type 1 diabetes*	Misto/Ensaio clínico randomizado não cego	Adolescentes com DM1^†^ em uso de SICI^‡^ e seus pais
A*10	Quiró, et al., 2019^25^	*Experiences and real life management of insulin pump therapy in adults with type 1 diabetes*	Misto/Estudo transversal	Adultos com DM1^†^ em uso do SICI^‡^
A*11	Grando et al., 2017^26^	*Characterization of Exercise and Alcohol Self-Management Behaviors of Type 1 Diabetes Patients on Insulin Pump Therapy*	Qualitativo	Adultos com DM1^†^ em uso do SICI^‡^
A*12	Groat, et al., 2017^27^	*Self-Management Behaviors in Adults on Insulin Pump Therapy*	Quantitativo/Coorte	Adultos com DM1^†^ em uso do SICI^‡^
A*13	Martyn-Nemeth, et al., 2017^28^	*Fear of hypoglycemia: Influence on glycemic variability and self-management behavior in young adults with type 1 diabetes*	Quantitativo/Estudo Prospectivo	Adultos com DM1^†^ em uso do SICI^‡^
A*14	Olinder, et al., 2011^29^	*Clarifying responsibility for self-management of diabetes in adolescents using insulin pumps--a qualitative study*	Qualitativo	Adolescentes com DM1^†^ em uso do SICI^‡^, pais e enfermeira pediátrica de diabetes
A*15	Wilson, 2008^30^	*Barriers to effective communication between patients using insulin pump therapy technology to enable intensive diabetes self-management and the health professionals providing their diabetes care*	Misto	Adultos com DM1^†^ em uso do SICI^‡^
A*16	Berlin, et al., 2006^31^	*Contextual Assessment of Problematic Situations Identified by Insulin Pump Using Adolescents and Their Parents*	Qualitativo	Adolescentes com DM1^†^ em uso do SICI^‡^ e pais
A*17	Ritholz, et al., 2007^32^	*Perceptions of Psychosocial Factors and the Insulin Pump*	Qualitativo	Adultos com DM1^†^ em uso do SICI^‡^
A*18	Weissberg-Benchell, et al., 2017^33^	*The use of Continuous Subcutaneous Insulin Infusion (CSII): parental and professional perceptions of self-care mastery and autonomy in children and adolescents*	Qualitativo	Pais de crianças e adolescentes com DM1^†^ em uso do SICI^‡^ e médicos de diabetes

*A=Artigo; ^†^DM1 = Diabetes mellitus tipo 1; ^‡^SICI = Sistema de Infusão Contínua de Insulina

### Síntese dos resultados

Os resultados foram divididos em duas categorias, quais sejam: 1) Comportamentos de autocuidado e 2) Fatores que influenciam o autocuidado. Foram elencados três comportamentos de autocuidados: Manejo glicêmico, Administração de *bolus* e Contagem de carboidratos; e cinco fatores que influenciam o autocuidado: Conhecimento; Liberdade e flexibilidade; Uso do sensor de monitoramento de glicose; Contexto e Responsabilidade (Figura 4).

**Figura 4:** Classificação do autocuidado das pessoas com DM1* em uso do SICI^†^. Fortaleza, CE, Brasil, 2025

*DM1 = Diabetes Mellitus Tipo 1; ^†^SICI = Sistema de Infusão Contínua de Insulina

### Comportamentos de autocuidado

#### Monitoramento glicêmico

O aumento da frequência do automonitoramento da glicose capilar (AMGC) foi associado à diminuição da hemoglobina glicada (HbA1c) em pessoas com DM1 em uso do SICI^22^ ^,^ ^25^ ^,^ ^33^. Além da frequência do AMGC, adolescentes que registraram uma leitura de glicose antes de cada refeição apresentaram HbA1c significativamente menor do que os adolescentes que não o fizeram^22^.

#### Administração de bolus

O aumento da frequência de *bolus* de insulina do SICI foi associado à diminuição da HbA1c em pessoas com DM1^22^ ^,^ ^25^ ^,^ ^33^. Aumentar o número de *bolus* diários de insulina teve o maior impacto no aumento do número de leituras de glicose capilar na meta para o dia (r = 0,93)^27^.

Estudos examinaram o momento da administração do *bolus* de insulina, além da frequência da administração do *bolus* ^22^ ^,^ ^24^ ^-^ ^25^ ^,^ ^27^. Identificou-se que o *bolus* noturno teve o maior efeito na HbA1c com a administração de todos os *bolus* noturnos associada à redução da HbA1c de 43,2 (21,6-64,7) mmol/mol [5,59% (IC 95%: 2,79%, 8,37%)] em comparação com crianças que não realizaram *bolus* nas refeições noturnas^22^.

Nesse sentido, alguns autores^25^ ^-^ ^33^ relataram que a calculadora de *bolus* tem sido utilizada com frequência entre as pessoas em uso do SICI. Estudo^33^ demonstrou que 79% dos pacientes em uso do SICI usavam a calculadora todos os dias. Ao considerar o número total de *bolus*/dia, outro estudo identificou que a maior parte das pessoas em uso do SICI utilizou a calculadora de *bolus* em comparação ao cálculo do *bolus* pelo sistema manual (20,9%)^25^.

O uso da calculadora de *bolus* de insulina tem sido associado a melhores níveis de HbA1c. Estudo^22^ realizado nos EUA identificou que os adolescentes que sempre usaram a calculadora de *bolus* da bomba de insulina tiveram HbA1c significativamente menor e eram mais propensos a atingir uma meta de HbA1c≤7,5%, quando comparados a adolescentes que usaram a calculadora de bolus menos de 50% do tempo (respectivamente, 7,8 ± 0,2% vs. 8,5%, p = 0,015; 67,9 ± 19,4% vs. 30,8 ± 7,1%, p = 0,0731).

#### Contagem de carboidratos

Observou-se que a contagem de carboidratos estava presente quando avaliados os comportamentos de autocuidado de pessoas com DM1 em uso do SICI^19^ ^,^ ^24^ ^-^ ^27^.

Aponta-se que pacientes em uso do SICI tinham uma alta frequência em inserir os carboidratos na dieta, três ou mais vezes/dia, com média de 76,6%. Isso mostra que o uso do SICI possibilita à pessoa um controle diário de aporte de carboidratos, favorecendo uma maior efetividade nesse comportamento. Talvez, porque deve-se inserir a quantidade de carboidratos ingeridos na calculadora de bolus^27^.

Os níveis de adesão à contagem de carboidratos foram semelhantes entre aqueles no monitoramento contínuo da glicose (29,7%) e no monitoramento da glicemia capilar (33,3%). Cinco participantes tiveram adesão a esse comportamento 100% do tempo, enquanto apenas um participante mostrou um máximo de duas entradas de carboidratos por dia. Os carboidratos foram documentados em média 3,9 vezes por dia^27^.

O número total de carboidratos por dia foi maior (141±58 vs. 121±64g/dia; p < 0,05) entre os participantes em uso do SICI, os quais apresentavam HbA1c ≤7,5%^25^. Esse dado mostra que, quanto mais frequentes são os registros de carboidratos no dispositivo, melhor tende a ser o controle glicêmico do paciente. 

### Fatores que influenciam o autocuidado

#### Conhecimento

Pacientes e profissionais de saúde necessitam adquirir conhecimento da terapia com SICI para usá-lo efetivamente e administrar insulina segundo a necessidade. Os estudos analisados abordaram o conhecimento e o autocuidado em duas dimensões, quais sejam: conhecimento da pessoa em uso do SICI ou do profissional de saúde^20^ ^-^ ^22^ e necessidade de suporte sobre conhecimento^23^ ^-^ ^30^.

Adolescentes que utilizam o SICI e com maior conhecimento sobre o DM1 tendem a relatar maior independência na autogestão da doença em comparação com aqueles com conhecimento mais limitado^22^. Um entendimento aprofundado sobre a sua condição e de seu tratamento facilita uma autogestão mais eficaz e segura. 

Nesse contexto, um estudo^20^ realizado com usuários do SICI, utilizando simulação realística, identificou erros de autogestão durante a simulação. Dentre eles, destacaram-se a escolha de alimentos sem carboidratos para tratar ou prevenir a hipoglicemia, a falta de verificação de cetonas, a omissão na troca do local de infusão da bomba e a falha em prevenir a hipoglicemia por meio do consumo de carboidratos ou da redução temporária da basal antes do exercício. 

A carência de informações sobre o tratamento com SICI e a falta de suporte no tratamento ficaram evidentes nos estudos analisados^23^ ^-^ ^30^. Usuários de SICI entrevistados em um dos estudos revelaram que não receberam apoio dos profissionais de saúde para o autogerenciamento intensivo do diabetes, nem para o uso do SICI^30^.

No mesmo estudo^30^, os usuários de SICI perceberam que seu tratamento não era centrado no atendimento de suas necessidades, sendo que 88,1% dos participantes relataram que receberam explicações inadequadas dos profissionais de saúde. Além disso, grande parte (91,0%) disse que suas necessidades de cuidados e suporte para o controle da doença não foram atendidas devido à falta de treinamento profissional de saúde no uso da terapia com SICI.

A educação em diabetes centrada na pessoa contribui para melhorar os níveis de HbA1C, e a educação personalizada pode atender às expectativas de pessoas com DM1 que necessitam de suporte individualizado^21^.

#### Liberdade e flexibilidade

Os participantes apreciaram a liberdade e a flexibilidade fornecidas pelo tratamento com SICI^21^ ^-^ ^32^. Após ultrapassar as dificuldades para iniciar o tratamento, eles sentiram que tinham a liberdade de viver suas vidas como desejavam e frequentemente ajustavam seu tratamento à sua situação atual^21^.

As pessoas elegiam uma programação básica para a vida diária, mas mudavam com frequência para outros programas, dependendo das atividades do dia, como: doses de *bolus* eram frequentemente usadas, conforme alimentação e uso de sensores para melhores ajustes no tratamento. Além do mais, expressaram que o uso do dispositivo levou a uma melhora da HbA1C de níveis anteriores^21^.

Ao analisar o autocuidado de pessoas em uso do SICI, estudo realizado com grupos de pacientes com níveis de HbA1c baixa, média e alta, identificou-se que todos os grupos consideraram que o uso da bomba de insulina é uma das ferramentas que permite o autocuidado mais “conveniente”, expresso em termos de ter maior “flexibilidade” e “liberdade”^32^.

Os membros do grupo com níveis de HbA1c baixa, média e alta se descreveram como ativamente envolvidos em seu autocuidado com o diabetes. Declararam que, além da “conveniência”, o verdadeiro benefício da bomba era o manejo glicêmico aprimorado. Por outro lado, participantes do grupo A1C alto focaram principalmente na “conveniência” do SICI e expressaram relutância em realizar comportamentos de autocuidado esperados, como não valorizar registros alimentares ou diários de glicemia, e admitiram às vezes esquecer de usar insulina em *bolus* ^32^.

#### Responsabilidade

Os estudos^29^ ^-^ ^33^ que abordaram a temática responsabilidade e autogestão do DM1 foram realizados com adolescentes e pais. Um dos estudos^29^ apontou que a falta de responsabilidade parece ser a principal razão para doses de *bolus* perdidas e autogestão insuficiente.

A responsabilidade pode ser explicada por meio de três subcategorias: Distribuição de responsabilidade, Transferência de responsabilidade e Esclarecimento de responsabilidade^29^.

A Distribuição da responsabilidade pelo autogerenciamento do diabetes é clara entre aqueles que usam a maioria de suas doses em *bolus*. Alguns desses adolescentes disseram que seus pais não sabiam muito sobre o tratamento com SICI e, por isso, não queriam sua ajuda. Eles queriam cuidar de si mesmos. Como o tratamento com SICI é mais tecnológico do que injeções, muitos adolescentes manuseiam as bombas com mais facilidade do que os pais. Isso faz com que os pais retirem sua responsabilidade, deixando-os realizar seu autocuidado^29^.

A Transferência ótima de responsabilidade é quando gradualmente passa dos pais para o adolescente. Eles podem precisar de mais suporte quando estão doentes ou quando a glicemia está muito alta ou baixa ou de difícil controle. Às vezes, os adolescentes podem assumir mais responsabilidade por um tempo, podem fazer mais coisas sozinhos sem perder o controle sobre o diabetes^29^.

Adolescentes e pais precisam negociar e esclarecer a responsabilidade pelo autogerenciamento do diabetes. Essa negociação precisa ser realizada continuamente, e as equipes de tratamento do DM1 podem dar suporte a essas negociações. Os pais precisam saber o que podem fazer para facilitar a autogestão dos adolescentes^29^.

#### Uso do sensor de monitoramento de glicose

O sistema de monitoramento de glicose *flash* (FGM) possibilita ao paciente uma forma fácil para monitorar a glicose do fluido intersticial, de forma contínua, proporcionando um autocuidado mais ativo^16^. 

Estudo^16^ realizado com pacientes em uso do SICI constatou que após 12 semanas em uso do FGM, as atividades de autocuidado que apresentaram grau de significância maior de mudança foram : 1) Gestão da glicose: os itens “verificar meus níveis de açúcar no sangue com cuidado e atenção” (p=0,041) e “registrar os níveis de açúcar no sangue regularmente” (p=0,032); 2) Controle dietético: os itens “Os alimentos que escolho comer facilitam a obtenção de níveis ideais de açúcar no sangue” (p=0,024) e “Ocasionalmente, como muitos doces ou outros alimentos ricos em carboidratos (p=0,049)”; 3) Uso em cuidados de saúde: os itens “Tenho tendência a evitar consultas médicas relacionadas com a diabetes” (p=0,031) e “Meu autocuidado com diabetes é ruim” (p=0,021).

Além disso, os pacientes com um maior número de exames diários de FGM revelaram níveis significativamente melhorados de HbA1c^16^.

### Contexto

Um dos estudos^31^, realizado com adolescentes em uso do SICI e seus pais, identificou a autogestão como um domínio em que, segundo eles, apresentavam dificuldade. Uma imagem diferente de autogestão surgiu, no entanto, quando o contexto foi considerado. Tanto mães quanto pais relataram dificuldades com autogestão mais frequentemente dentro de um contexto familiar ou dentro de múltiplos contextos, enquanto os jovens relataram mais dificuldades frequentes de autogestão em um contexto de pares. 

Ao considerar o agrupamento geral das situações mais frequentemente relatadas, diferentes padrões emergem para cada membro da família. Para os jovens, a interação modal de conteúdo e contexto foi dificuldade de autogerenciamento dentro de um contexto social, enquanto para as mães foi autogerenciamento dentro de um contexto familiar. Os pais, por outro lado, relataram dois domínios de conteúdo modal (hipoglicemia e mau funcionamento da bomba), ambos ocorrendo com mais frequência dentro de um contexto familiar^31^.

## Discussão

O autocuidado em pacientes com DM1 em uso do SICI é fundamental para garantir um controle glicêmico eficaz, reduzir complicações agudas e crônicas e promover qualidade de vida. O SICI oferece benefícios significativos, mas exige comprometimento e conhecimento por parte do paciente para que seu uso seja seguro e eficaz.

Nesse sentido, o monitoramento glicêmico é uma peça-chave no sucesso do tratamento com o SICI, sendo que, na dimensão do autocuidado, envolve interpretar os dados e ajustar a dose de insulina quando necessário (refeições, exercícios, correções).

Para um manejo glicêmico adequado da pessoa com DM1, são necessários uso contínuo de doses de insulina, automonitoramento da glicemia, acompanhamento com profissionais qualificados para suporte e educação em saúde e um estilo de vida saudável^34^.

O ideal é que o automonitoramento seja realizado de sete a nove vezes ao dia, de acordo com a terapêutica e necessidade, sendo uma vez em jejum ao acordar, todas as vezes antes da refeição (pré-prandial), duas horas após a refeição (pós-prandial), e no horário de dormir^35^.

A equipe de saúde e o paciente devem estar capacitados e engajados para lidar com as causas e os efeitos obtidos no automonitoramento. Quando o paciente está envolvido nesse comportamento de autocuidado, a possibilidade de melhora clínica é ainda maior, pois esta é uma eficiente ferramenta de motivação para ele^35^.

Vivenciar o DM1 constitui-se uma experiência desafiadora, que gera conflitos e dificuldades diante da imprevisibilidade da doença, devido às exigências e mudanças no estilo de vida requeridas pelo tratamento. A partir do diagnóstico, o cuidado pode se tornar complexo, pois envolve diversas tarefas diárias que afetam a dinâmica familiar, além de obstáculos na adesão ao tratamento, influenciados por fatores clínicos e sociodemográficos que podem impactar o estado de saúde^36^. Portanto, a educação em diabetes, por parte da equipe de saúde multidisciplinar capacitada, é importante no acolhimento da família e do paciente.

Nesse contexto, é fundamental disponibilizar recursos tecnológicos aos profissionais de saúde, para que possam atuar como facilitadores em processos de cuidado e educação, ajudando os pacientes com DM1 a reavaliar comportamentos e pensamentos, a fim de promover cuidados de saúde mais eficazes^37^. 

No tratamento com SICI a reposição da insulina exógena pode ser realizada de diferentes formas, tais como insulina basal; insulina *bolus* pré-refeições e insulina com a finalidade de corrigir hiperglicemias pré e pós-prandiais ou no período entre as refeições^38^.

Em alguns modelos de SICI existem diferentes tipos de *bolus*, que podem ser administrados imediatamente ou por um período maior, dependendo das necessidades do paciente em um determinado momento^39^. Por um lado, a insulina é liberada continuamente e programada (taxa basal) para controlar a produção hepática de glicose durante o período noturno e pós-prandial tardio (> 4 horas). E, por outro lado, é liberada sob demanda (*bolus*) da pessoa antes das refeições (*bolus* prandial) ou para correção da hiperglicemia (*bolus* de correção)^38^.

A maioria dos modelos de SICI apresenta uma calculadora de *bolus* e realiza o cálculo para a sua infusão. O paciente precisa apenas informar o valor da glicemia e se vai ingerir carboidratos (CH), colocando a quantidade em gramas ou porções (1 porção = 10 g de CH)^40^. Isso facilita a aplicação de insulina, já que a pessoa não precisa fazer cálculos.

Alguns aplicativos de gerenciamento alimentar possuem opção de cálculo automático da dose de insulina. Ainda é desafio para muitas pessoas com DM1 estimar a dose apropriada de insulina. A dificuldade de calcular doses adequadas de insulina pode ser o motivo pelo qual muitas pessoas não atingem seus objetivos terapêuticos, expresso por um nível elevado de hemoglobina glicada^41^.

Implementar uma dieta para manter os parâmetros nutricionais dentro do desejado tem se mostrado um dos maiores desafios para o tratamento das pessoas com diabetes. Para realizar um plano alimentar personalizado, uma das principais condutas a serem implementadas para pessoas com DM1 é a contagem de carboidratos. Baseia-se na quantidade de carboidrato total que a pessoa pode ou deve consumir por dia, segundo sua rotina, seus horários e medicamentos^42^.

Um estudo brasileiro^43^ avaliou fatores preditivos do controle glicêmico em crianças e adolescentes com DM1 e os resultados indicaram que a duração da doença e a dose de insulina estavam diretamente associadas a níveis mais elevados de HbA1c, enquanto o uso da contagem de carboidratos estava associado a uma redução na HbA1c.

Muitas pessoas com DM1 podem ser candidatas ao uso do SICI. No entanto, para que o tratamento seja bem-sucedido, é fundamental que esteja motivada, consciente e tenha conhecimento técnico e capacidade de autogestão. Além disso, é essencial que receba treinamento adequado e seja continuamente monitorada para garantir o uso correto do sistema^44^.

Em relação ao conhecimento sobre o SICI, os estudos encontrados nesta revisão abordaram e identificaram que, quanto maior o conhecimento por parte dos pacientes e profissionais, melhor o manejo e segurança do tratamento.

Eventos adversos podem ser detectados e prevenidos usando estratégias de solução de problemas, o que ressalta a importância da educação do paciente ao iniciar a terapia com SICI^45^. Além disso, alguns erros e eventos adversos podem acontecer pela falta de conhecimento.

Nesse contexto, é intuitivo considerar que o controle eficaz do DM1 depende não apenas do uso adequado dos medicamentos, mas também do conhecimento dos pacientes sobre seu tratamento, da adoção de hábitos alimentares saudáveis, da prática de exercícios e do automonitoramento da glicemia^46^.

O SICI vem se mostrando como um método eficaz no manejo glicêmico, proporcionando maior comodidade ao usuário e maior liberdade para suas tarefas diárias. Está associada à melhora dos níveis de HbA1c e menor taxa de hipoglicemia noturna. No entanto, a pessoa precisa estar motivada a aprender sobre os métodos disponíveis no SICI para atingir o controle glicêmico^47^.

Com o aumento das responsabilidades após o diagnóstico de DM1, os jovens precisam, junto com seus responsáveis, gerenciar o autocuidado, o que resulta em uma maior responsabilidade desde cedo diante dos desafios diários. É fundamental que essas pessoas recebam apoio, especialmente da família, para evitar uma sobrecarga emocional que possa comprometer seu autocuidado^48^.

A aquisição da responsabilidade de autocuidado pelos adolescentes é geralmente apoiada pelo apoio contínuo dos pais e começa com uma interdependência entre as tarefas de gerenciamento do diabetes. Há uma transição natural nas responsabilidades de cuidado à medida que os adolescentes começam a se afastar da família e passar mais tempo em atividades acadêmicas, esportivas, sociais e até mesmo atividades relacionadas ao trabalho^48^.

Revisões sistemáticas^49^ ^-^ ^50^ identificaram que o monitoramento contínuo da glicose (CGM) era fácil de usar e melhorava o empoderamento do paciente e a autonomia no gerenciamento dos níveis de glicose, prevenindo a hipoglicemia.

Corroborando esses achados, vários estudos^51^ ^-^ ^53^ indicaram que a frequência do automonitoramento está conectada com níveis de HbA1c melhorados e redução de complicações relacionadas ao diabetes devido à associação direta entre o monitoramento diário e o controle do diabetes. 

Os resultados deste estudo apontam que a importância do autocuidado de indivíduos com DM1 em uso do SICI se pauta nos comportamentos de autocuidado e nos fatores que influenciam esse autocuidado, melhoria da qualidade de vida dos pacientes e minimização de complicações de saúde.

Apesar das contribuições importantes desta revisão para o campo científico e a prática, há limitações. Embora seja uma metodologia robusta para a síntese de evidências em saúde, essa revisão de escopo, diferentemente da revisão sistemática tradicional, não incorpora uma avaliação da qualidade metodológica ou um cálculo combinado do efeito das intervenções. Consequentemente, os estudos incluídos neste trabalho não foram avaliados e sintetizados considerando o seu nível de evidência.

No que se refere às lacunas encontradas, foi observado que há pouca evidência sobre o autocuidado de pessoas com DM1 em uso do SICI. Logo, essas questões ressaltam a necessidade premente de pesquisas adicionais, bem como de colaboração interdisciplinar entre profissionais da saúde para avaliação do autocuidado dessa população específica, em diferentes faixas etárias, e melhor compreensão desse fenômeno e suas necessidades nas diferentes fases da vida.

## Conclusão

Em síntese, esta revisão proporcionou uma visão abrangente sobre o autocuidado de pessoas com DM1 em uso de SICI, destacando os comportamentos e os fatores que influenciam o autocuidado. Diante disso, entende-se que compreender o autocuidado no manejo do DM1 por meio do SICI é essencial, dada sua complexidade e as exigências contínuas do tratamento. 

Vale destacar que um conhecimento aprofundado sobre o autocuidado permite aos profissionais atualização e uma melhor tomada de decisão quanto ao plano terapêutico individualizado, e às pessoas com DM1, buscar informações e ações sobre os cuidados diários para o adequado manejo do diabetes, especialmente o manuseio seguro do SICI. Destaca-se a necessidade de estudos futuros de intervenção que otimizem o autocuidado nessa população.

## Data Availability

Todos os dados gerados ou analisados durante este estudo estão incluídos neste artigo publicado.

# Translation: Sistema de Infusión Continua de Insulina y autocuidado en la Diabetes Mellitus Tipo 1: revisión de alcance

**DOI:** 10.1590/1518-8345.8001.4851

**Authors:** Raquel Rodrigues da Costa Brilhante, Carla Cristina de Sordi, Paulo Weslen Carneiro Gonçalves, Vanessa de Araujo Lima Freire, Amelina de Brito Belchior, Maria Luiza Pereira Costa, Raquel Sampaio Florêncio, Sherida Karanini Paz de Oliveira

**Affiliations:** 1 Universidade Estadual do Ceará, Fortaleza, CE, Brasil.

**Keywords:** Insulina, Sistemas de Infusión de Insulina, Autocuidado, Autogestión, Conocimiento, Diabetes Mellitus

## Abstract

**Objetivo:**

mapear las evidencias disponibles en la literatura sobre el autocuidado de personas con Diabetes Mellitus Tipo 1 que utilizan el Sistema de Infusión Continua de Insulina.

**Método:**

se trata de una revisión de alcance basada en los principios recomendados por el JBI y en las directrices PRISMA para la extracción de datos, realizada a partir de diez bases de datos y da literatura gris. Se utilizó la estrategia PCC para la recopilación de datos. La selección de los estudios se realizó tras la eliminación de duplicados, la evaluación individual y por pares. Se utilizaron análisis descriptivos y diagramas para presentar los resultados.

**Resultados:**

un análisis de los 18 artículos seleccionados mostró que existen tres comportamientos de autocuidado: manejo glucémico, bolo de insulina y recuento de carbohidratos. Los factores que influyen en estos comportamientos fueron el conocimiento, la libertad/flexibilidad, el uso del sensor, el contexto y la responsabilidad.

**Conclusión:**

los estudios demuestran que un conocimiento profundo sobre el autocuidado permite a los profesionales tomar mejores decisiones respecto al plan terapéutico individualizado; y las personas que utilizan el Sistema de Infusión Continua de Insulina buscan acciones e información en relación con el cuidado diario, para un manejo adecuado de la diabetes. Se destaca la necesidad de futuros estudios de intervención que optimicen el autocuidado en esta población.

## Destacados

**(1)** Identifica los comportamientos de autocuidado de las personas con DM1. **(2)** Analiza los factores que influyen en los comportamientos de autocuidado. **(3)** Ayuda a los profesionales a tomar decisiones sobre el plan terapéutico individualizado. **(4)** Ayuda a los profesionales a buscar acciones e información con relación a los cuidados diarios.

## Introducción

La Diabetes Mellitus tipo 1 (DM1) es una enfermedad autoinmune caracterizada por la destrucción de las células beta en el páncreas, que suele conducir a una deficiencia absoluta de insulina^1^. Aunque es el tipo de diabetes mellitus más común en niños y adolescentes, los investigadores indican que hay más casos nuevos de DM1 diagnosticados en la vida adulta que en la infancia y adolescencia^2^.

En 2022, se estimó que había 8,75 millones de personas con diabetes tipo 1 en todo el mundo. Brasil fue uno de los diez países con el mayor número de casos prevalentes de DM1 en todas las edades^3^. Este escenario epidemiológico requiere estrategias que fomenten prácticas de autocuidado para lograr resultados terapéuticos satisfactorios y prevenir complicaciones. Las prácticas de autocuidado incluyen la planificación de la alimentación, la actividad física, el uso regular de medicamentos, especialmente la insulina, y la comprobación rutinaria de glucosa capilar^4^.

Para un manejo glucémico satisfactorio de DM1, se enfatiza un tratamiento intensivo de terapia con insulina, que debe imitar la secreción fisiológica de insulina^5^. Entre estos tratamientos, el Sistema de Infusión Continua de Insulina (SICI) es un dispositivo electrónico que infunde insulina a través del tejido subcutáneo de forma ininterrumpida, similar a lo que ocurre en el cuerpo de una persona sin diabetes, para intentar obtener euglucemia entre comidas y liberar insulina durante las comidas. El uso de la SICI puede proporcionar mayor seguridad y autonomía en relación con el tratamiento de DM1^6^.

Para utilizar la SICI, la persona con diabetes debe adquirir y desarrollar conocimientos y habilidades para la atención adecuada con el tratamiento. Para ello, se necesita un enfoque interprofesional con un enfoque en la educación sobre la diabetes para la atención al paciente. Los dispositivos para la diabetes, como el SICI, son máquinas complejas que dependen en gran medida de la competencia individual, la vigilancia y los comportamientos de autogestión para lograr beneficios clínicos^7^. Por ello, es importante evaluarlas y monitorizarlas constantemente para garantizar buenos resultados glucémicos y de salud.

Existe una extensa literatura científica^8^ ^-^ ^10^ sobre la diabetes mellitus (DM) y numerosos estudios relacionados con el autocuidado. Sin embargo, hasta la fecha, no se ha realizado ningún intento previo de establecer un análisis del autocuidado de personas con DM1 utilizando el SICI.

Al considerar la relevancia y las especificidades de la atención para personas con enfermedades crónicas, especialmente aquellas con diabetes tipo 1 que utilizan SICI, la importancia de las investigaciones sobre prácticas de autocuidado y sus factores intermedios, así como formas de estimular estas prácticas y ofrecer una atención cualificada y eficaz, este estudio pretende mapear la evidencia disponible en la literatura sobre el autocuidado de personas con DM1 utilizando un Sistema de Infusión Continua de Insulina.

## Método

### Protocolo y registro

Revisión de alcance basada en el *Manual for Evidence Synthesis* (Manual para la Síntesis de Evidencia), propuesto por el Instituto Joanna Briggs (JBI)^11^, siguiendo los siguientes pasos: (1) pregunta de investigación, (2) criterios de elegibilidad, (3) estrategia de búsqueda, (4) extracción de datos, (5) síntesis y presentación de los principales hallazgos. Los autores siguieron las recomendaciones del *checklist Preferred Reporting Items for Systematic and Meta-Analyses - Extension for Scoping Reviews* (PRISMA-ScR)^12^.

Esta es una recomendación de revisión bibliográfica utilizada para mapear los conceptos principales, resumir la evidencia, permitir una mayor amplitud de la literatura e informar futuras investigaciones que incluyen la preparación de un borrador de revisión, la elaboración de criterios de inclusión, la estrategia de búsqueda, la extracción, la presentación y resumen de resultados y sus implicaciones para la investigación y la práctica^11^.

Este tipo de revisión se adoptó para explorar la evidencia disponible sobre el autocuidado de personas con DM1 usando SICI, permitiendo así apreciar este atributo. El protocolo de revisión fue registrado en el *Open Science Framework* (OSF) bajo el DOI: 10.17605/OSF.IO/A3U9E.

La estrategia PCC (Población, Concepto y Contexto) se utilizó para construir la pregunta de investigación, siendo la población (P) personas con diabetes mellitus tipo 1, el concepto (C) abordado era el autocuidado y el contexto (C) el uso del SICI, lo que resultó en la siguiente pregunta principal orientadora: ¿Cuál es la evidencia científica sobre el autocuidado de personas con diabetes mellitus tipo 1 utilizando el Sistema de Infusión Continua de Insulina?

### Criterios de elegibilidad

Los criterios de elegibilidad se definieron según la estrategia PCC: (1) Población (P), personas diagnosticadas con DM1, de cualquier edad, de ambos sexos, con o sin comorbilidades; (2) Concepto (C), estudios con un enfoque de actividades de autocuidado para personas con DM1 utilizando el SICI; estudios que describían el proceso de evaluación de estas actividades; y estudios que evaluaron dos o más actividades de autocuidado; y (3) Contexto (C), estudios sobre el uso de cualquier marca/modelo de SICI. 

En cuanto al tipo de estudio, se eligieron estudios primarios y secundarios, que podían ser cuantitativos, cualitativos o mixtos, de cualquier diseño o metodología, que consideraran el autocuidado realizado por personas con DM1 utilizando el SICI. Además, se consideraron estudios publicados completos, en formato completo, nacionales o internacionales, en cualquier idioma, sin límite temporal.

Los estudios que involucraron, además de personas con DM1 usando el SICI, otros participantes, como profesionales sanitarios o familiares, solo considerarían datos que se refieren a personas con DM1 que usaban el SICI.

Se excluyeron estudios con personas con diabetes mellitus tipo 2 o diabetes gestacional utilizando el SICI, protocolos de investigación, artículos de opinión, editoriales, cartas al director y resúmenes en anales de eventos, mientras que no existen resultados concisos sobre las actividades de autocuidado de personas con DM1 usando el SICI.

### Fuentes de información

La búsqueda tuvo lugar el 2 de octubre de 2024, utilizando el acceso institucional del portal de la revista CAPES/CAFe *Access* (Comunidad Académica), cuando no hay acceso gratuito y gratuito. Así, la búsqueda se realizó en las siguientes bases de datos/portal: *Scientific Electronic Library Online* (SciELO), PubMed, Scopus (Elsevier), *Medical Literature Analysis and Retrieval System Online* (MEDLINE) vía VHL, *Web of Science*, *ScienceDirect*, *Cumulative Index to Nursing and Allied Health Literature* (CINAHL), *Latin American and Caribbean Literature in Health Sciences* (LILACS) vía VHL, *Cochrane Library* y Embase.

La literatura gris fue recuperada de cuatro fuentes: *Google Scholar*, Biblioteca Digital Brasileña de Tesis y Disertaciones (BDBTD), Catálogo de Tesis y Disertaciones (CTD) de la Coordinación para la Mejora del Personal de Educación Superior (CAPES), Tesis y Disertaciones de Acceso Abierto (OATD). La recuperación de los documentos en *Google Scholar* se realizó en las primeras diez páginas, sin filtro de periodo.

### Búsqueda

La estrategia de búsqueda se construyó basándose en los Descriptores de Ciencias de la Salud (DeCS), *Medical Subject Headings* (MeSH), EMTREE y lenguaje natural junto con los operadores booleanos AND y OR. Se guió por la estrategia ECUS^13^ (Extracción, Conversión, Combinación, Construcción y Uso), que presenta paso a paso para la formulación de una estrategia de búsqueda sensible cuyas etapas son: extracción, conversión, combinación, construcción y uso (Figura 1).

**Figura 1:** Estrategia ECUS* para la formulación de la estrategia de búsqueda. Fortaleza, CE, Brasil, 2025

Etapas	Estrategia ECUS*		
	P^†^	C^‡^	C^§^
Extracción	Personas con diabetes mellitus tipo 1	Autocuidado	Sistemas de Infusión de Insulina
Conversión	Diabetes mellitus tipo 1	Self Care	Insulin Infusion Systems
Combinación	Diabetes mellitus type 1	Self Care; Self-Care; Self management	Insulin Infusion Systems; Insulin Pump
Construcción	“Diabetes Mellitus Type 1” OR “Diabetes Mellitus, Type 1” OR “Diabetes, Type 1” OR “IDDM^||^” OR “Type 1 Diabetes” OR “Type 1 Diabetes Mellitus”	Self Care” OR “Self-Care”OR “Self-management”	“Insulin infusion systems” OR “Insulin pump”
Uso	“Diabetes Mellitus Type 1” OR “Diabetes Mellitus, Type 1” OR “Diabetes, Type 1” OR “IDDM” OR “Type 1 Diabetes” OR “Type 1 Diabetes Mellitus” AND “self care” OR “Self-care” OR “Self-management” AND “nsulin infusion systems” OR “Insulin pump”		

*ECUS = Extracción, Conversión, Combinación, Construcción y Uso; ^†^P = Población; ^‡^C = Concepto; ^§^C = Contexto; ^||^IDDM = *Insulin-Dependent Diabetes Mellitus*

Con la definición realizada y probada, los términos y palabras clave se adaptaron a cada base de datos, utilizando como modelo la estrategia de búsqueda elaborada para la PubMed/MEDLINE: “Diabetes Mellitus type 1” OR “Diabetes Mellitus, Type 1” OR “Diabetes, Type 1” OR “IDDM” OR “Type 1 Diabetes” OR “Type 1 Diabetes Mellitus” AND “Self Care” OR “Self-care” OR “Self-management” AND “Insulin Infusion Systems” OR “Insulin Pump”.

### Selección de fuentes de evidencias

Los estudios obtenidos en las bases fueron importados al gestor de referencias *Rayyan* ^®^ versión *web* gratuita, que permite el análisis automático y manual de duplicados. Con la ayuda de esta herramienta, dos investigadores pudieron realizar la evaluación de forma ciega e independiente en dos etapas: (1) lectura del título y resumen y (2) lectura del texto completo. La selección y cribado de los estudios fue realizada de forma independiente por dos investigadores, RRCB y CCS. Cualquier desacuerdo se resolvería mediante un tercer revisor (SKPO), que no fue activado. Según la literatura, los desacuerdos pueden resolverse por consenso o mediante un tercer revisor^14^.

Tras eliminar los duplicados, los artículos se seleccionaron leyendo los títulos y resúmenes, basándose en los criterios preestablecidos en el estudio. Por ello, los estudios se leyeron íntegros para verificar la permanencia.

Los datos de los artículos incluidos se extrajeron utilizando una hoja de cálculo de Microsoft Excel^*®*^ , que contenía información específica sobre título, autores, año, país de origen, enfoque metodológico, objetivo del estudio, población del estudio, número de participantes, intervenciones, resultados principales y referencia. 

### Síntesis de los resultados

Tras extraer los datos, se utilizó análisis de contenido, de tipo temático-categórico, cuando se descubren los núcleos de significado de esa comunicación cuya frecuencia o presencia tiene un significado para el objetivo del estudio^15^.

### Aspectos éticos

Como se trata de un estudio de revisión de alcance, se exime su aprobación por parte del Comité de Ética en Investigación.

## Resultados

### Selección de fuentes de evidencias

La investigación resultó inicialmente en 1125 publicaciones en las bases de datos y 107 en la literatura gris, sumando un total de 1232. Fueron excluidas 572 por ser duplicados, quedando 660 para la lectura del título y el resumen. Tras el primer análisis, con la lectura de títulos y resúmenes revisados, se excluyeron 638 y se seleccionaron 22 para la lectura completa. De estos, tres fueron excluidos porque no respondieron a la pregunta de investigación y uno fue duplicado. La muestra final consistió en 18 artículos (Figura 2).

**Figura 2:** Diagrama de flujo del proceso de selección de estudios adaptado del *Preferred Reporting Items for Systematic Review and Meta-Analyses extension for Scoping Reviews* (PRISMA-ScR*)^12^

*PRISMA-ScR = *Preferred Reporting Items for Systematic reviews and Meta-Analyses extension for Scoping Reviews*; ^†^SciELO = *Scientific Electronic Library Online*; ^‡^MEDLINE = *Medical Literature Analysis and Retrieval System Online*; ^§^BVS = Biblioteca Virtual en Salud; ^||^LILACS = Literatura Latinoamericana y del Caribe en Ciencias de la Salud; ^¶^CINAHL = *Cumulative Index to Nursing and Allied Health Literature*; ^**^SICI = Sistema de Infusión Continua de Insulina

### Características de las fuentes de evidencias

La Figura 3 presenta la caracterización de los artículos que se incluyeron como muestra final de esta revisión de alcance. Las publicaciones se publican entre 2006 y 2023, con un aumento significativo en los últimos 10 años, ya que se publicaron 14 estudios (73,6%) en este periodo.

En cuanto al idioma, los artículos se publicaron en inglés y portugués (n=18)^16^ ^-^ ^33^. El país con más publicaciones sobre el tema fue Estados Unidos (n=10)^18^ ^,^ ^20^ ^,^ ^22^ ^,^ ^24^ ^,^ ^26^ ^-^ ^28^ ^,^ ^31^ ^-^ ^33^, seguido de Suecia^21^ ^-^ ^29^ e Inglaterra^23^ ^-^ ^30^ con dos artículos cada uno, y después Arabia Saudí^16^, Brasil^17^, Dinamarca^19^ y España^25^ con una publicación cada uno (Figura 3). 

En cuanto al tipo de estudio, la mayoría se definieron como cuantitativos (n=3)^16^ ^,^ ^27^ ^-^ ^28^, seguidos de cualitativos (n=9)^17^ ^,^ ^21^ ^-^ ^23^ ^,^ ^26^ ^,^ ^29^ ^,^ ^31^ ^-^ ^33^ y mixtos (n=6)^18^ ^-^ ^20^ ^,^ ^24^ ^-^ ^25^ ^,^ ^30^. Se realizaron once estudios con adultos que usaban SICI^16^ ^-^ ^17^ ^,^ ^19^ ^-^ ^21^ ^,^ ^25^ ^-^ ^28^ ^,^ ^30^ ^,^ ^32^; tres estudios con adolescentes y padres^22^ ^,^ ^24^ ^,^ ^31^, un estudio con adolescentes utilizando el SICI, padres y enfermeros pediátricos de diabetes^29^, uno con padres de niños y adolescentes usando el SICI y médicos de diabetes^33^ y un estudio con adultos que usaban el SICI y 20 profesionales sanitarios especialistas^23^. Cabe destacar que solo se utilizaron datos que se refieren a personas con DM1 que usan SICI.

Los resultados relacionados con el autocuidado de personas con DM1 que usaban SICI se identificaron en los artículos seleccionados; Por ello, para describirlo de forma sistemática, se realizó un análisis del contenido abordado, identificando en el texto las características más citadas mediante la categorización.

**Figura 3:** Caracterización de los estudios incluidos en la revisión de alcance, según autor/referencia, año, título, enfoque metodológico/diseño del estudio y población estudiada. Fortaleza, CE, Brasil, 2025

Artículos	Autor/Año	Título	Enfoque metodológico/diseño de estudio	Población estudiada
A*1	Al Hayek, 2021^16^	*Effectiveness of the freestyle libre 2 flash glucose monitoring system on diabetes-self-management practices and glycemic parameters among patients with type 1 diabetes using insulin pump*	Cuantitativo/de cohorte prospectivo	Adultos con DM1^†^ usando SICI^‡^
A*2	Brandão, et al., 2023^17^	*Aplicativos para autogestão do diabetes tipo 1 em usuários de sistema de infusão contínua de insulina*	Cualitativo/Revisión integrativa	Usuarios de SICI^‡^ con DM1^†^
A*3	Faulds, et al., 2021^18^	*Expect the unexpected: Adolescent and pre-teens’ experience of diabetes technology self-management*	Mixto	Adolescentes con DM1^†^ usando SICI^‡^
A*4	Rytter, et al., 2023^19^	*Associations between insulin pump self-management and HbA1c in type 1 diabetes*	Mixto/Estudio Observacional	Adultos con DM1^†^ usando SICI^‡^
A*5	Faulds, et al., 2022^20^	*Simulation platform development for diabetes and technology self-management*	Mixto/Estudio Observacional	Adultos con DM1^†^ usando SICI^‡^
A*6	Persson, et al., 2022^21^	*‘Striving for freedom or remaining with what is well-known’: a focus-group study of self-management among people with type 1 diabetes who have suboptimal glycaemic control despite continuous subcutaneous insulin infusion*	Cualitativo	Adultos con DM1^†^ usando SICI^‡^
A*7	Faulds, et al., 2021^22^	*State of the science: A scoping review and gap analysis of adolescent insulin pump self-management*	Cualitativo	Adolescentes con DM1^†^ usando SICI^‡^
A*8	Reidy, Foster, Rogers, 2020^23^	*A facilitated web-based self-management tool for people with type 1 diabetes using an insulin pump: intervention development using the behavior change wheel and theoretical domains framework*	Cualitativo	Adultos con DM1^†^ usando SICI^‡^ y profesionales de la salud especialistas
A*9	O’Donnell, et al., 2021^24^	*“Pump It Up! A randomized clinical trial to optimize insulin pump self-management behaviors in adolescents with type 1 diabetes*	Mixto/Ensayo clínico aleatorizado no ciego	Adolescentes con DM1^†^ usando SICI^‡^ y sus padres
A*10	Quiró, et al., 2019^25^	*Experiences and real life management of insulin pump therapy in adults with type 1 diabetes*	Mixto/Estudio transversal	Adultos con DM1^†^ usando SICI^‡^
A*11	Grando et al., 2017^26^	*Characterization of Exercise and Alcohol Self-Management Behaviors of Type 1 Diabetes Patients on Insulin Pump Therapy*	Cualitativo	Adultos con DM1^†^ usando SICI^‡^
A*12	Groat, et al., 2017^27^	*Self-Management Behaviors in Adults on Insulin Pump Therapy*	Cuantitativo/Cohorte	Adultos con DM1^†^ usando SICI^‡^
A*13	Martyn-Nemeth, et al., 2017^28^	*Fear of hypoglycemia: Influence on glycemic variability and self-management behavior in young adults with type 1 diabetes*	Cuantitativo/Estudio Prospectivo	Adultos con DM1^†^ usando SICI^‡^
A*14	Olinder, et al., 2011^29^	*Clarifying responsibility for self-management of diabetes in adolescents using insulin pumps--a qualitative study*	Cualitativo	Adolescentes con DM1^†^ usando SICI^‡^, padres y enfermera pediátrica especialista en diabetes
A*15	Wilson, 2008^30^	*Barriers to effective communication between patients using insulin pump therapy technology to enable intensive diabetes self-management and the health professionals providing their diabetes care*	Mixto	Adultos con DM1^†^ usando SICI^‡^
A*16	Berlin, et al., 2006^31^	*Contextual Assessment of Problematic Situations Identified by Insulin Pump Using Adolescents and Their Parents*	Cualitativo	Adolescentes con DM1^†^ usando SICI^‡^ y padres
A*17	Ritholz, et al., 2007^32^	*Perceptions of Psychosocial Factors and the Insulin Pump*	Cualitativo	Adultos con DM1^†^ usando SICI^‡^
A*18	Weissberg-Benchell, et al., 2017^33^	*The use of Continuous Subcutaneous Insulin Infusion (CSII): parental and professional perceptions of self-care mastery and autonomy in children and adolescents*	Cualitativo	Padres de niños y adolescentes con DM1^†^ usando SICI^‡^ y médicos de diabetes

*A=Artículo; ^†^DM1 = Diabetes mellitus tipo 1; ^‡^SICI = Sistema de Infusión Continua de Insulina

### Síntesis de los resultados

Los resultados se dividieron en dos categorías, a saber: 1) Conductas de autocuidado y 2) Factores que influyen en el autocuidado. Se enumeraron tres comportamientos de autocuidado: manejo glucémico, administración de bolos y recuento de carbohidratos; y cinco factores que influyen en el autocuidado: Conocimiento; Libertad y flexibilidad; Uso del sensor de monitorización de glucosa; Contexto y responsabilidad (Figura 4).

**Figura 4:** Clasificación del autocuidado de personas con DM1* usando SICI^†^. Fortaleza, CE, Brasil, 2025

*DM1 = Diabetes Mellitus Tipo 1; ^†^SICI = Sistema de Infusión Continua de Insulina

### Conductas de autocuidado

#### Monitorización glucémica

El aumento en la frecuencia de la automonitorización capilar de la glucosa (AMGC) se asoció con una disminución de la hemoglobina glicalizada (HbA1c) en personas con DM1 utilizando SICI^22^ ^,^ ^25^ ^,^ ^33^. Además de la frecuencia de la AMGC, los adolescentes que registraron una lectura de glucosa antes de cada comida tenían HbA1c significativamente más baja que los adolescentes que no lo hicieron^22^.

#### Administración de bolos

El aumento de la frecuencia de bolos de insulina del SICI se asoció con una disminución de la HbA1c en personas con DM1^22^ ^,^ ^25^ ^,^ ^33^. Aumentar el número de bolos de insulina diarios tuvo el mayor impacto en aumentar el número de lecturas capilares de glucosa en el objetivo del día (r = 0,93)^27^.

Se han realizado estudios para examinar el momento de la administración del bolo de insulina, además de la frecuencia de la administración del bolo^22^ ^,^ ^24^ ^-^ ^25^ ^,^ ^27^. Se descubrió que el bolo nocturno tenía el mayor efecto sobre la HbA1c, y que la administración de todos los bolos nocturnos se asociaba con una reducción de la HbA1c de 43,2 (21,6-64,7) mmol/mol [5,59% (IC del 95%: 2,79%, 8,37%)] en comparación con los niños que no recibieron bolos en las comidas nocturnas^22^.

En este sentido, algunos autores^25^ ^-^ ^33^ informaron que la calculadora de bolus se ha utilizado frecuentemente entre personas que usan el SICI. Un estudio^33^ demostró que el 79% de los pacientes que usaban la SICI utilizaban la calculadora todos los días. Considerando el número total de bolos al día, otro estudio identificó que la mayoría de las personas que usaban la SICI usaban la calculadora de bolos en comparación con los bolos manuales (20,9%)^25^.

El uso de la calculadora de bolo de insulina se ha asociado con mejores niveles de HbA1c. Un estudio^22^ realizado en EE. UU. identificó que los adolescentes que siempre usaron la calculadora de bolos de la bomba de insulina tenían HbA1c significativamente más bajo y tenían más probabilidades de alcanzar un objetivo de HbA1c≤7,5%, en comparación con los adolescentes que usaron la calculadora de bolos menos del 50% de las veces (respectivamente, 7,8 ± 0,2% frente a 8,5%, p = 0,015; 67,9 ± 19,4% frente a 30,8 ± 7,1%, p = 0,0731).

#### Recuento de carbohidratos

Se observó que el recuento de carbohidratos estaba presente al evaluar los comportamientos de autocuidado de personas con DM1 utilizando SICI^19^ ^,^ ^24^ ^-^ ^27^.

Se señala que los pacientes que usaban el SICI tenían una alta frecuencia de inserción de carbohidratos, tres o más veces al día, con una media del 76,6%. Esto demuestra que el uso del SICI permite a la persona controlar la ingesta diaria de carbohidratos, favoreciendo una mayor eficacia en este comportamiento. Quizá porque la cantidad de carbohidratos ingeridos debe introducirse en la calculadora de bolos^27^.

Los niveles de adherencia al recuento de carbohidratos fueron similares entre los que se realizaban el monitoreo continuo de glucosa (29,7%) y el monitoreo capilar de glucosa (33,3%). Cinco participantes mostraron adherencia a este comportamiento el 100% del tiempo, mientras que solo uno mostró un máximo de dos insumos de carbohidratos al día. Se han documentado carbohidratos de media 3,9 veces al día^27^.

El número total de carbohidratos por día fue mayor (141±58 frente a 121±64g/día; p < 0,05) entre los participantes que usaron el SICI, que tenían HbA1c ≤7,5%^25^. Estos datos demuestran que cuanto más frecuentes sean los registros de carbohidratos en el dispositivo, mejor tiende a ser el control glucémico de este paciente.

### Factores que influyen en el autocuidado

#### Conocimiento

Los pacientes y profesionales de la salud deben tener conocimientos sobre la terapia SICI para utilizarla eficazmente y administrar insulina según sea necesario. Los estudios analizados abordaron el conocimiento y el autocuidado en dos dimensiones, a saber: el conocimiento de la persona que utiliza el SICI o del profesional sanitario^20^ ^-^ ^22^ y la necesidad de apoyo al conocimiento^23^ ^-^ ^30^.

Los adolescentes que utilizan la SICI y tienen mayor conocimiento sobre la diabetes tienden a reportar mayor independencia en la autogestión de la enfermedad en comparación con aquellos con conocimientos más limitados^22^. Un conocimiento profundo de la enfermedad y su tratamiento facilita una autogestión más eficaz y segura.

En este contexto, un estudio^20^ realizado con usuarios de SICI, utilizando simulación realista, identificó errores de autogestión durante la simulación. Estas incluían elegir alimentos sin carbohidratos para tratar o prevenir la hipoglucemia, la falta de control de cetonas, la falta de cambio de lugar de infusión de la bomba y la falta de prevención de la hipoglucemia mediante el consumo de carbohidratos o la reducción temporal de la línea basal antes del ejercicio. 

La falta de informaciones sobre el tratamiento con SICI y la falta de apoyo en el tratamiento fueron evidentes en los estudios analizados^23^ ^-^ ^30^. Los usuarios de SICI entrevistados en uno de los estudios revelaron que no recibieron apoyo de profesionales sanitarios para la autogestión intensiva de la diabetes, ni para el uso de SICI^30^.

En el mismo estudio^30^, los usuarios de SICI percibieron que su tratamiento para la diabetes no estaba centrado en el paciente para satisfacer sus necesidades, ya que el 88,1% de los participantes informó que recibieron explicaciones insuficientes por parte de los profesionales sanitarios. Además, una gran parte (91,0%) afirmó que sus necesidades de atención y apoyo para la diabetes no se cubrieron debido a la falta de formación profesional sanitaria en el uso de la terapia SICI.

La educación sobre diabetes centrada en la persona contribuye a mejorar los niveles de HbA1C, y la educación personalizada puede satisfacer las expectativas de las personas con DM1 que necesitan apoyo individualizado^21^.

#### Libertad y flexibilidad

Los participantes valoraron la libertad y flexibilidad que ofrece el tratamiento SICI^21^ ^-^ ^32^. Tras las dificultades iniciales para iniciar el tratamiento, sentían que tenían la libertad de vivir sus vidas como quisieran y a menudo ajustaban su tratamiento a su situación actual^21^.

Las personas elegían un horario básico para la vida diaria, pero con frecuencia cambiaba a otros programas, dependiendo de las actividades del día, como: dosis de bolo se usaban con frecuencia, según la dieta, y el uso de sensores para mejores ajustes en el tratamiento. Además, expresaron que el uso del dispositivo condujo a una mejora en la HbA1C respecto a niveles anteriores^21^.

Al analizar el autocuidado de personas que usan SICI, un estudio realizado con grupos de pacientes con HbA1c bajo, medio y alto identificó que todos los grupos discutían la bomba de insulina como que hace el autocuidado más “conveniente”, expresado en términos de mayor “flexibilidad” y “libertad”^32^.

Los miembros del grupo de HbA1c bajo, medio y alto se describieron como activos en el autocuidado de su diabetes. Afirmaron que, aparte de la “comodidad”, el verdadero beneficio de la bomba era una mejor gestión glucémica. Por otro lado, los participantes del grupo con A1C alto se centraron principalmente en la “conveniencia” del SICI y expresaron reticencia a realizar comportamientos de autocuidado esperados, como no valorar los registros de alimentos o los diarios de glucosa en sangre, y admitieron que a veces se olvidaban de usar insulina en bolo^32^.

#### Responsabilidad

Los estudios^29^ ^-^ ^33^ que abordaron el tema de la responsabilidad y la autogestión de la diabetes se llevaron a cabo con adolescentes y padres. Uno de los estudios^29^ señaló que la falta de responsabilidad parece ser la principal causa de las dosis de bolos perdidas y la autogestión insuficiente.

La responsabilidad puede explicarse a través de tres subcategorías: Distribución de la responsabilidad, Transferencia de responsabilidad y Clarificación de la responsabilidad^29^.

La distribución de la responsabilidad en la autogestión de la diabetes es clara entre quienes utilizan la mayoría de sus dosis de bolos. Algunos de estos adolescentes dijeron que sus padres no sabían mucho sobre el tratamiento de la SICI, así que no querían su ayuda. Querían cuidarse a sí mismos. Como el tratamiento de SICI es más tecnológico que las inyecciones, muchos adolescentes manejan las bombas con más facilidad que sus padres. Esto hace que los padres retiren su responsabilidad, permitiéndoles cuidarse a sí mismos^29^.

La transferencia óptima de responsabilidad ocurre cuando pasa gradualmente de los padres al adolescente. Puede necesitar más apoyo cuando está enferma, cuando su glucosa en sangre está demasiado alta, baja o es difícil de controlar. A veces, los adolescentes pueden asumir más responsabilidades durante un tiempo, pueden hacer más cosas por sí mismos sin perder el control sobre la diabetes^29^. 

Los adolescentes y los padres deben negociar y aclarar la responsabilidad de la autogestión de la diabetes. Esta negociación debe hacerse de forma continua, y los equipos de atención a la diabetes pueden apoyar estas negociaciones. Los padres deben saber qué pueden hacer para facilitar la autogestión de los adolescentes^29^.

#### Uso de sensores de monitoramento de glucosa

El sistema de monitorización de glucosa *flash* (FGM) proporciona al paciente una forma sencilla de monitorizar la glucosa del líquido intersticial de forma continua, proporcionando un autocuidado más activo^16^.

Un estudio^16^ realizado con pacientes que usaban SICI encontró que tras 12 semanas de MGF, se produjeron las actividades de autocuidado que presentaron un mayor grado de significado de cambio: 1) Gestión de la glucosa: los ítems “comprobar mis niveles de azúcar en sangre de forma cuidadosa y atenta” (p=0,041) y “registrar los niveles de azúcar en sangre regularmente” (p=0,032); 2) Control dietético: los elementos “Los alimentos que elijo comer facilitan alcanzar niveles óptimos de azúcar en sangre” (p=0,024) y “Ocasionalmente, como muchos dulces u otros alimentos ricos en carbohidratos (p=0,049)”; 3) Uso en la atención sanitaria: los elementos “Tiendo a evitar citas médicas relacionadas con la diabetes” (p=0,031) y “Mi autocuidado de la diabetes es pobre” (p=0,021).

Además, los pacientes con un mayor número de exámenes diarios de FGM mostraron niveles significativamente mejorados de HbA1c^16^.

### Contexto

Uno de los estudios^31^, realizado con adolescentes que usaban SICI y sus padres, identificó la autogestión como un dominio que, según ellos, presentaba dificultades. Sin embargo, surgió una imagen diferente de la autogestión al considerar el contexto. Tanto madres como padres informaron de dificultades para la autogestión con mayor frecuencia dentro de un contexto familiar o en múltiples contextos, mientras que los jóvenes reportaron dificultades más frecuentes para la autogestión en un contexto de compañeros.

Al considerar la agrupación general de las situaciones más frecuentemente reportadas, emergen diferentes patrones para cada miembro de la familia. Para los jóvenes, la interacción modal entre contenido y contexto era dificultad para la autogestión dentro de un contexto social, mientras que para las madres era la autogestión dentro de un contexto familiar. Los padres, por otro lado, reportaron dos dominios de contenido modal (hipoglucemia y mal funcionamiento de la bomba), ambos ocurriendo con mayor frecuencia dentro de un contexto familiar^31^.

## Discusión

El autocuidado en pacientes con DM1 que utilizan SICI es esencial para garantizar un control glucémico eficaz, reducir complicaciones agudas y crónicas, y promover la calidad de vida. El SICI ofrece beneficios significativos, pero requiere compromiso y conocimiento por parte del paciente para que su uso sea seguro y eficaz.

En este sentido, el seguimiento glucémico es una pieza clave para el éxito del tratamiento con SICI, y en el ámbito del autocuidado, implica interpretar los datos y ajustar la dosis de insulina cuando sea necesario (comidas, ejercicios, correcciones).

Para un manejo glucémico adecuado de las personas con DM1, es necesario el uso continuo de dosis de insulina, el autocontrol de la glucosa en sangre, el seguimiento con profesionales cualificados para apoyo y educación sanitaria, y un estilo de vida saludable^34^.

Idealmente, la autoevaluación debería realizarse entre siete y nueve veces al día, según la terapia y la necesidad, una vez en ayuno al despertarse, cada vez antes de una comida (preprandial), 2 horas después de una comida (postprandial) y a la hora de dormir^35^. 

El equipo de la salud y el paciente deben estar formados y comprometidos para abordar las causas y efectos que se obtienen en la automonitorización. Cuando el paciente está involucrado en este comportamiento de autocuidado, la posibilidad de mejora clínica es aún mayor, ya que para él es una herramienta de motivación eficiente^35^.

Experimentar DM1 es una experiencia desafiante, que genera conflictos y dificultades debido a la imprevisibilidad de la enfermedad, a las exigencias y cambios en el estilo de vida que requiere el tratamiento. A partir del diagnóstico, la atención puede volverse compleja, ya que implica varias tareas diarias que afectan a la dinámica familiar, además de obstáculos en la adhesión al tratamiento, influenciados por factores clínicos y sociodemográficos que pueden influir en el estado de salud^36^. Por lo tanto, la educación diabetológica, impartida por un equipo multidisciplinario de salud capacitado, es importante para dar la bienvenida a la familia y al paciente.

En este contexto, es esencial ofrecer recursos tecnológicos a los profesionales sanitarios, para que puedan actuar como facilitadores en los procesos de atención y educación, ayudando a los pacientes con DM1 a reevaluar comportamientos y pensamientos, con el fin de promover una atención sanitaria más eficaz^37^.

En el tratamiento con SICI, la reposición de insulina exógena puede realizarse de diferentes formas, tales como insulina basal; insulina en bolo antes de las comidas e insulina con el fin de corregir hiperglucemias pre y posprandiales o en el período entre las comidas^38^.

En algunos modelos de SICI, existen diferentes tipos de bolos, que pueden administrarse de inmediato o durante un periodo prolongado, dependiendo de las necesidades del paciente en un momento dado^39^. Por un lado, la insulina se libera continuamente y se programa (tasa basal) para controlar la producción hepática de glucosa durante la noche y el periodo postprandial (> 4 horas). Y, por otro lado, se libera bajo demanda (bolo) de la persona antes de las comidas (bolo prandial) o para corregir la hiperglucemia (bolo de corrección)^38^.

La mayoría de los modelos SICI cuentan con una calculadora de bolus y realizan el cálculo para la infusión de bolus. El paciente solo necesita informar el valor de glucosa en sangre y si ingerirá carbohidratos (CH), introduciendo la cantidad en gramos o porciones (1 porción = 10 g de CH)^40^. Esto facilita la aplicación de insulina, ya que la persona no necesita hacer cálculos.

Algunas aplicaciones de gestión de alimentos tienen una opción para calcular automáticamente la dosis de insulina. Sigue siendo un reto para muchas personas con DM1 estimar la dosis adecuada de insulina. La dificultad para calcular dosis adecuadas de insulina puede ser la razón por la que muchas personas no alcanzan sus objetivos terapéuticos, expresados por un alto nivel de hemoglobina glicada^41^.

Implementar una dieta que mantenga los parámetros nutricionales dentro del rango deseado ha resultado ser uno de los mayores retos para el tratamiento de las personas con diabetes. Para llevar a cabo un plan de alimentación personalizado, una de las principales conductas que deben aplicarse en personas con DM1 es el recuento de carbohidratos. Se basa en la cantidad total de carbohidratos que la persona puede o debe consumir al día, según su rutina, horarios y medicación^42^.

Un estudio brasileño^43^ evaluó factores predictivos de control glucémico en niños y adolescentes con DM1 y los resultados indicaron que la duración de la enfermedad y la dosis de insulina se asociaron directamente con niveles más altos de HbA1c, mientras que el uso del recuento de carbohidratos se asoció con una reducción de HbA1c.

Muchas personas con diabetes tipo 1 pueden ser candidatas para el uso del SICI. Sin embargo, para que el tratamiento tenga éxito, es esencial que estén motivados, sean conscientes y tengan conocimientos técnicos y habilidades de autogestión. Además, es esencial que reciban una formación adecuada y sean monitorizados continuamente para garantizar el correcto uso del sistema^44^.

En cuanto al conocimiento sobre el SICI, los estudios encontrados en esta revisión abordaron e identificaron que cuanto mayor es el conocimiento por parte de pacientes y profesionales, mejor será la gestión y la seguridad del tratamiento.

Los eventos adversos pueden detectarse y prevenirse mediante estrategias de resolución de problemas, lo que pone de manifiesto la importancia de la educación del paciente al iniciar la terapia con SICI^45^. Además, pueden ocurrir algunos errores y eventos adversos debido a la falta de conocimiento.

En este contexto, resulta intuitivo considerar que el control efectivo de DM1 depende no solo del uso adecuado de los medicamentos, sino también del conocimiento que tienen los pacientes sobre su tratamiento, la adopción de hábitos alimentarios saludables, la práctica de ejercicios y el autocontrol de la glucemia^46^.

Se ha demostrado que el SICI es un método eficaz para el manejo glucémico, proporcionando mayor comodidad al usuario y mayor libertad para sus tareas diarias. Está asociada a una mejora en los niveles de HbA1c y a una menor tasa de hipoglucemia nocturna. Sin embargo, la persona debe estar motivada para aprender sobre los métodos disponibles en el SICI para lograr el control glucémico^47^.

Con el aumento de responsabilidades tras el diagnóstico de DM1, los jóvenes necesitan, junto con sus tutores, gestionar el autocuidado, lo que resulta en una mayor responsabilidad desde una edad temprana ante los desafíos diarios. Es esencial que estas personas reciban apoyo, especialmente de la familia, para evitar una sobrecarga emocional que pueda comprometer su autocuidado^48^.

La adquisición de la responsabilidad de autocuidado por parte de los adolescentes suele estar apoyada por el apoyo continuo de los padres y comienza con una interdependencia entre las tareas de manejo de la diabetes. Existe una transición natural en las responsabilidades de cuidado a medida que los adolescentes comienzan a alejarse de sus familias y dedican más tiempo a actividades académicas, deportivas, sociales e incluso relacionadas con el trabajo^48^.

Las revisiones sistemáticas^49^ ^-^ ^50^ encontraron que el monitoreo continuo de glucosa (CGM) era fácil de usar y mejoraba el empoderamiento y la autonomía del paciente en el manejo de los niveles de glucosa, previniendo la hipoglucemia.

Corroborando estos hallazgos, varios estudios^51^ ^-^ ^53^ han indicado que la frecuencia del autocontrol está asociada con una mejora en los niveles de HbA1c y una reducción de las complicaciones relacionadas con la diabetes debido a la asociación directa entre el seguimiento diario y el control de la diabetes.

Los resultados de este estudio indican que la importancia del autocuidado para las personas con DM1 que utilizan el SICI se basa en los comportamientos de autocuidado y en los factores que influyen en este autocuidado, mejorando la calidad de vida de los pacientes y minimizando las complicaciones de salud.

A pesar de las importantes contribuciones de esta revisión al campo y la práctica científica, presenta limitaciones. Aunque es una metodología robusta para la síntesis de evidencia en salud, esta revisión de alcance, a diferencia de la revisión sistemática tradicional, no incorpora una evaluación de la calidad metodológica ni un cálculo combinado del efecto de las intervenciones. En consecuencia, los estudios incluidos en este estudio no fueron evaluados ni sintetizados teniendo en cuenta su nivel de evidencia.

En cuanto a las lagunas encontradas, se observó que hay poca evidencia sobre el autocuidado de personas con DM1 utilizando el SICI. Por ello, estas preguntas ponen de manifiesto la urgente necesidad de investigación adicional, así como de una colaboración interdisciplinar entre profesionales sanitarios para evaluar el autocuidado de esta población específica, en diferentes grupos de edad, y una mejor comprensión de este fenómeno y sus necesidades en las distintas etapas de la vida.

## Conclusión

En resumen, esta revisión proporcionó una visión general sobre el autocuidado de las personas con diabetes tipo 1 que utilizan SICI, destacando los comportamientos y los factores que influyen en el autocuidado. En vista de ello, se entiende que comprender el autocuidado en el manejo de la DM1 mediante SICI es esencial, dada su complejidad y las exigencias continuas del tratamiento.

Cabe destacar que un conocimiento profundo sobre el autocuidado permite a los profesionales actualizar y tomar mejores decisiones respecto al plan terapéutico individualizado; y personas con DM, para buscar información y acciones sobre el cuidado diario para el manejo adecuado de la diabetes, especialmente el manejo seguro del SICI. Se destaca la necesidad de futuros estudios de intervención que optimicen el autocuidado en esta población.

## Data Availability

Todos los datos generados o analizados durante este estudio están incluidos en este artículo publicado.
